# Cortico-basal ganglia white matter microstructure is linked to restricted repetitive behavior in autism spectrum disorder

**DOI:** 10.1186/s13229-023-00581-2

**Published:** 2024-01-23

**Authors:** Bradley J. Wilkes, Derek B. Archer, Anna L. Farmer, Carly Bass, Hannah Korah, David E. Vaillancourt, Mark H. Lewis

**Affiliations:** 1 Department of Applied Physiology and Kinesiology, University of Florida, P.O. Box 118205, Gainesville, FL 32611 USA; 2 Vanderbilt Memory and Alzheimer’s Center, Department of Neurology, Vanderbilt School of Medicine, Nashville, TN USA; 3 Department of Neurology, Vanderbilt Genetics Institute, Vanderbilt School of Medicine, Nashville, TN USA; 4 Department of Psychology, University of Florida, Gainesville, FL USA; 5 Department of Psychiatry, University of Florida, Gainesville, FL USA; 6 Department of Pharmacology, College of Medicine, University of Arizona, Tucson, AZ USA; 7 Department of Biomedical Engineering, University of Florida, Gainesville, FL USA; 8 Department of Neurology, Fixel Center for Neurological Diseases, Program in Movement Disorders and Neurorestoration, University of Florida, Gainesville, FL USA

**Keywords:** Autism spectrum disorder, Restricted repetitive behavior, Diffusion tensor imaging, Free-water, Basal ganglia, Cerebellum, Cortico-basal ganglia, Gray matter, White matter

## Abstract

**Background:**

Restricted repetitive behavior (RRB) is one of two behavioral domains required for the diagnosis of autism spectrum disorder (ASD). Neuroimaging is widely used to study brain alterations associated with ASD and the domain of social and communication deficits, but there has been less work regarding brain alterations linked to RRB.

**Methods:**

We utilized neuroimaging data from the National Institute of Mental Health Data Archive to assess basal ganglia and cerebellum structure in a cohort of children and adolescents with ASD compared to typically developing (TD) controls. We evaluated regional gray matter volumes from T1-weighted anatomical scans and assessed diffusion-weighted scans to quantify white matter microstructure with free-water imaging. We also investigated the interaction of biological sex and ASD diagnosis on these measures, and their correlation with clinical scales of RRB.

**Results:**

Individuals with ASD had significantly lower free-water corrected fractional anisotropy (FA_T_) and higher free-water (FW) in cortico-basal ganglia white matter tracts. These microstructural differences did not interact with biological sex. Moreover, both FA_T_ and FW in basal ganglia white matter tracts significantly correlated with measures of RRB. In contrast, we found no significant difference in basal ganglia or cerebellar gray matter volumes.

**Limitations:**

The basal ganglia and cerebellar regions in this study were selected due to their hypothesized relevance to RRB. Differences between ASD and TD individuals that may occur outside the basal ganglia and cerebellum, and their potential relationship to RRB, were not evaluated.

**Conclusions:**

These new findings demonstrate that cortico-basal ganglia white matter microstructure is altered in ASD and linked to RRB. FW in cortico-basal ganglia and intra-basal ganglia white matter was more sensitive to group differences in ASD, whereas cortico-basal ganglia FA_T_ was more closely linked to RRB. In contrast, basal ganglia and cerebellar volumes did not differ in ASD. There was no interaction between ASD diagnosis and sex-related differences in brain structure. Future diffusion imaging investigations in ASD may benefit from free-water estimation and correction in order to better understand how white matter is affected in ASD, and how such measures are linked to RRB.

**Supplementary Information:**

The online version contains supplementary material available at 10.1186/s13229-023-00581-2.

## Background

Autism spectrum disorder (ASD) includes two diagnostic domains: (1) social and communication deficits and (2) restricted repetitive behavior (RRB). RRB refers to multiple categories of repeating patterns of behavior that often occur with high frequency, have no clear function, interfere with appropriate behavior, and can sometimes result in bodily harm. Although many research and intervention efforts in ASD have targeted social and communication deficits, there has been considerably less focus on the RRB domain. Although behavioral interventions have some efficacy in treating RRB [1–3], pharmacological interventions have no demonstrated efficacy for treating RRB in ASD, but rather are used primarily to treat associated problems (e.g., aggressive behavior) [4, 5]. This lack of efficacious treatments is largely due to an incomplete understanding of the neural circuitry mediating RRB in ASD. There is a pressing need to elucidate how RRB relates to differences in brain structure and function in ASD.

There has been increasing focus on hypotheses regarding brain connectivity in ASD, and how disrupted connectivity patterns relate to the two diagnostic domains. Investigations of connectivity differences in ASD have largely utilized magnetic resonance imaging (MRI) to assess structural and functional connectivity. Functional MRI and diffusion tensor imaging (DTI) in ASD both generally support reduced connectivity within major brain networks, though there is also evidence of increased connectivity between networks [6–10]. Although connectivity differences in ASD have frequently been studied in the context of social and communication deficits [11], far fewer studies have focused on altered connectivity as it pertains to RRB. [12]

Scalar measures from DTI provide insight into tissue microstructure and can be used to characterize structural aspects of brain connectivity. These scalar measures are confounded by partial volume effects from free-water (FW), such as cerebrospinal fluid. Using a two-compartment model, the unique contribution of FW can be quantified and other scalar measures adjusted accordingly [13]. The FW measure itself has already demonstrated significant value in a variety of neurodegenerative disorders and it is hypothesized that abnormal FW values are associated with atrophy and/or inflammation [14–19]. Despite the widespread use of DTI to study connectivity in ASD, FW quantification and correction have had limited application in this population [20, 21]. Many of the brain regions and white matter tracts hypothesized to be involved in the expression of RRB in ASD are proximal to the ventricular system, and as such, FW quantification may be of great value to DTI research in ASD.

In this study, we assessed gray matter volume and white matter microstructure in the basal ganglia and cerebellum in individuals with ASD compared to typically developing (TD) participants. We focused on the basal ganglia and cerebellum because they have been linked to RRB and ASD in prior work, yet these relationships remain poorly understood [12, 22–24]. We further investigated whether these measures differed between males and females with ASD, and whether they correlated with clinical measures of RRB. Current estimates suggest ASD is approximately four times more prevalent in males than females [25], and some imaging studies have suggested that the sex differences in brain morphology and connectivity in ASD are unlike those observed in TD individuals [26–31]. There is also mixed evidence of sex differences in the expression of RRB in ASD [32–36]. It remains unknown whether possible sex differences in the expression of RRB relate to sex differences in brain morphology or microstructure. In the present study, we used clinical and neuroimaging data available from the National Institute of Mental Health Data Archive (NDA) to investigate interactions between ASD diagnosis and biological sex on regional gray matter volumes in the basal ganglia and cerebellum, as well as microstructure in white matter pathways of the basal ganglia and cerebellum. We further investigated the relationship of these neuroimaging measures to clinical measures of RRB.

## Methods

### Participant data

Deidentified data were acquired from NDA (collection ID #2021). Several key features of this dataset guided our selection including the availability of Repetitive Behavior Scale-Revised scores on participants from both groups, adequate sample sizes, and a nearly equal number of males and females. For our purposes, selection criteria within this dataset included ages between 6 and 18 years, presence of T1-weighted anatomical and diffusion-weighted imaging data, and an intelligence quotient (IQ) measured with the Differential Ability Scales-II. We did not exclude individuals from this study based on IQ score. These data, however, were acquired retrospectively from the NDA and thus we did not have control over recruitment of participants with lower IQ scores. For the ASD group, inclusion also required diagnostic assessments: the Autism Diagnostic Observation Schedule-Second Edition (ADOS-2) and Autism Diagnostic Interview-Revised (ADI-R). Inclusion in the ASD group required an ADOS-2 score of 7 or higher. From the NDA dataset queried, 5 participants originally queried in the ASD dataset (4 males, 1 female) did not meet this cutoff and were excluded from subsequent analysis. Diagnostic assessments were not given to TD participants and thus were not required for inclusion in the TD group. Application of these initial criteria resulted in a dataset that included 91 individuals diagnosed with ASD (49 female) and 96 TD individuals (44 female). Details about the participants who met these criteria are included in Table 1.

**Table 1:** Demographic data and clinical scales

Group	*N*	Age in months (StDev)	IQ (StDev)	RBS-R Total (StDev)	ADI-R Section C (StDev)	SCQ (StDev)
*ASD*						
Male	42	146 (35.4)	102 (18.2)	23.0 (16.1)	6.46 (2.63)	20.4 (6.58)
Female	49	158 (31.9)	102 (22.3)	19.8 (17.4)	5.71 (2.55)	16.4 (7.57)
Total	91	152 (33.9)	102 (20.4)	21.3 (16.8)	6.03 (2.59)	18.3 (7.36)
*TD*						
Male	52	161 (32.5)	111 (16.2)	2.24 (6.61)	–	3.00 (3.69)
Female	44	157 (38.1)	109 (14.5)	0.98 (1.77)	–	1.77 (2.37)
Total	96	159 (35.0)	110 (15.4)	1.67 (5.07)	–	2.44 (3.20)

Number of participants in each group (ASD or TD) and subgroups of males and females, including means (± StDev) for age, IQ, RBS-R total score, ADI-R section C score, and SCQ score

Participants whose data met these initial inclusion criteria were then checked for quality of imaging data (e.g., no missing volumes or major motion artifacts). For anatomical scans, all remaining participants had adequate image quality and no major artifacts and thus were included in volumetric analysis. For diffusion-weighted images, we flagged diffusion volumes (i.e., directions) with more than 2 mm motion. A participant was excluded if more than 25% of their diffusion volumes exceeded this threshold. Applying these criteria, 15 participant’s data were excluded from diffusion MRI analysis (12 ASD, 3 TD).

### Magnetic resonance imaging parameters

Images from the broader collection ID (#2021) that these data were acquired from were collected across four sites: (1) The Center for Translational Developmental Neuroscience, Child Study Center, Yale School of Medicine, New Haven, CT; (2) The Nelson Laboratory of Cognitive Neuroscience, Boston Children’s Hospital, Harvard Medical School, Boston, MA; (3) The Center on Human Development & Disability, Seattle Children’s Hospital, University of Washington School of Medicine, Seattle, WA; (4) Staglin IMHRO Center for Cognitive Neuroscience, David Geffen School of Medicine, University of California, Los Angeles, CA. Scans were acquired on 3T Siemens scanners (TrioTim or Prisma) [28, 29]. The study dataset did not contain specific information about the collection site for each participant, but did contain details on the scanner type that each participant was scanned on. Thus, we covaried each imaging analyses by scanner type to control for this source of variability.

Anatomical images were acquired using a T1-weighted magnetization-prepared rapid acquisition gradient-echo (MPRAGE) sequence with the following parameters: 256 single-shot interleaved sagittal slices of 1 mm thickness; field of view (FOV) of 256 mm; 256 × 256 matrix; repetition time (TR) of 2530 ms; echo time (TE) of 3.31 ms (TrioTim scanners) or 3.34 ms (Prisma scanners), inversion time of 1100 ms; flip angle of 7º; bandwidth of 200 Hz/pixel; 100% phase and slice resolution.

Diffusion-weighted images were acquired using an echo-planar imaging sequence with the following parameters: 60 interleaved transverse slices of 2 mm thickness; 96 × 96 matrix; FOV of 190 mm; 96 × 96 matrix; TR of 9000 ms (TrioTim scanners) or 7300 ms (Prisma scanners), TE of 93 ms (TrioTim scanners) or 74 ms (Prisma scanners); echo spacing (*δ*) of 0.69 ms; 90º flip angle; bandwidth of 2264 Hz/pixel (TrioTim scanners) or 1680 Hz/pixel (Prisma scanners); 100% phase resolution. A diffusion weighting scheme with 64 diffusion directions at *b* = 1000 s/mm^2^ and one *b* = 0 s/mm^2^ volume was used.

### Data analysis

#### Demographic data and clinical scales

Participant age (in months) and IQ scores were compared using 2 × 2 analysis of variance (ANOVA) with diagnosis and sex as factors. Analysis of covariance (ANCOVA) was used to investigate effects of diagnosis and sex on repetitive behavior scores from the Repetitive Behavior Scale-Revised (RBS-R) [37], covarying for age. For the RBS-R, we assessed total score, as well as the following six subscale scores: stereotyped behavior, self-injurious behavior, compulsive behavior, ritualistic behavior, sameness behavior, and restricted interests. We also used ANCOVA to investigate effects of diagnosis and sex on social and communication scores from the lifetime version of the Social Communication Questionnaire (SCQ) [38], covarying for age.

For the ADI-R, scores were only available for individuals in the ASD group. Because the primary focus of this paper was RRB, we only performed statistical comparisons and neuroimaging correlations with scores from ADI-R section C, which pertain to RRB. We used a general linear model to explore the main effect of sex on ADI-R section C scores while covarying for age.

#### Basal ganglia and cerebellar volumes

We assessed basal ganglia and cerebellar volumes using ROIs from well-established atlases of the basal ganglia [39, 40] and cerebellum [41, 42]. We evaluated both left and right hemisphere ROIs in the basal ganglia and cerebellum, as well as midline structures in the cerebellum. We evaluated a total of 14 basal ganglia ROIs and 34 cerebellar ROIs (see Table 2).

**Table 2:** Basal ganglia and cerebellar volumes

Region of interest	% Total brain volume Mean ± Stdev		Diagnosis		Sex		Diagnosis * Sex	
	ASD	TD	*p*_raw_	*p*_FDR_	*p*_raw_	*p*_FDR_	*p*_raw_	*p*_FDR_
Caudate (*L*)	2.13E−01 ± 3.24E−02	2.15E−01 ± 3.41E−02	0.525	0.664	< 0.001	***< 0.001	0.427	0.558
Caudate (*R*)	2.00E−01 ± 3.17E−02	2.03E−01 ± 3.32E−02	0.283	0.467	< 0.001	***< 0.001	0.161	0.464
Putamen (*L*)	3.55E−01 ± 5.31E−02	3.66E−01 ± 5.09E−02	0.042	0.277	< 0.001	***< 0.001	0.020	0.238
Putamen (*R*)	3.45E−01 ± 5.34E−02	3.55E−01 ± 5.14E−02	0.036	0.277	< 0.001	***< 0.001	0.015	0.238
Nucleus accumbens (*L*)	7.94E−02 ± 1.12E−02	8.10E−02 ± 1.08E−02	0.126	0.301	< 0.001	***< 0.001	0.112	0.453
Nucleus accumbens (*R*)	7.65E−02 ± 1.10E−02	7.89E−02 ± 1.12E−02	0.025	0.277	< 0.001	***< 0.001	0.113	0.453
Globus pallidus external (*L*)	6.49E−02 ± 1.03E−02	6.63E−02 ± 9.75E−03	0.208	0.411	< 0.001	***< 0.001	0.017	0.238
Globus pallidus external (*R*)	5.98E−02 ± 9.80E−03	6.13E−02 ± 8.91E−03	0.150	0.327	< 0.001	***< 0.001	0.038	0.258
Globus pallidus internal (*L*)	2.36E−02 ± 4.07E−03	2.40E−02 ± 3.85E−03	0.271	0.465	< 0.001	***< 0.001	0.006	0.238
Globus pallidus internal (*R*)	2.20E−02 ± 3.69E−03	2.32E−02 ± 3.92E−03	0.019	0.277	< 0.001	***< 0.001	0.037	0.258
Subthalamic nucleus (*L*)	5.24E−03 ± 9.61E−04	5.36E−03 ± 1.10E−03	0.419	0.609	< 0.001	***< 0.001	0.139	0.464
Subthalamic nucleus (*R*)	5.19E−03 ± 9.53E−04	5.16E−03 ± 1.00E−03	0.804	0.839	< 0.001	***< 0.001	0.850	0.850
Substantia nigra (*L*)	1.19E−02 ± 1.81E−03	1.22E−02 ± 2.03E−03	0.292	0.467	0.003	**0.004	0.138	0.464
Substantia nigra (*R*)	1.20E−02 ± 1.81E−03	1.24E−02 ± 2.05E−03	0.112	0.301	< 0.001	***< 0.001	0.040	0.258
Lobule I−IV (*L*)	2.80E−01 ± 4.96E−02	2.82E−01 ± 4.75E−02	0.547	0.673	0.029	*0.031	0.381	0.558
Lobule I–IV (*R*)	2.91E−01 ± 5.22E−02	2.95E−01 ± 4.60E−02	0.459	0.623	0.020	*0.022	0.252	0.504
Lobule V (*L*)	3.30E−01 ± 5.39E−02	3.40E−01 ± 5.16E−02	0.113	0.301	< 0.001	**0.001	0.390	0.558
Lobule V (*R*)	3.37E−01 ± 5.82E−02	3.48E−01 ± 5.28E−02	0.105	0.301	0.006	**0.008	0.432	0.558
Lobule VI (*L*)	6.28E−01 ± 1.15E−01	6.60E−01 ± 8.94E−02	0.023	0.277	0.002	**0.003	0.510	0.627
Lobule VI (vermis)	1.51E−01 ± 3.19E−02	1.56E−01 ± 3.01E−02	0.230	0.425	0.002	**0.002	0.228	0.504
Lobule VI (*R*)	5.58E−01 ± 9.53E−02	5.86E−01 ± 9.04E−02	0.018	0.277	< 0.001	***< 0.001	0.523	0.627
Crus I (*L*)	1.02 ± 1.61E−01	1.05 ± 1.53E−01	0.069	0.277	< 0.001	***< 0.001	0.832	0.850
Crus I (vermis)	1.14E−03 ± 3.41E−04	1.16E−03 ± 3.55E−04	0.580	0.696	< 0.001	**0.001	0.113	0.453
Crus I (*R*)	1.02 ± 1.44E−01	1.06 ± 1.50E−01	0.038	0.277	< 0.001	***< 0.001	0.221	0.504
Crus II (L)	7.64E−01 ± 1.15E−01	7.70E−01 ± 1.35E−01	0.408	0.609	< 0.001	***< 0.001	0.292	0.540
Crus II (vermis)	3.63E−02 ± 7.70E−03	3.80E−02 ± 7.77E−03	0.062	0.277	< 0.001	**0.001	0.365	0.558
Crus II (*R*)	7.32E−01 ± 1.09E−01	7.49E−01 ± 1.23E−01	0.115	0.301	< 0.001	***< 0.001	0.105	0.453
Lobule VIIb (*L*)	3.57E−01 ± 5.39E−02	3.53E−01 ± 5.05E−02	0.859	0.877	< 0.001	***< 0.001	0.164	0.464
Lobule VIIb (vermis)	1.28E−02 ± 2.93E−03	1.34E−02 ± 2.66E−03	0.127	0.301	0.005	**0.006	0.539	0.631
Lobule VIIb (*R*)	3.81E−01 ± 5.49E−02	3.80E−01 ± 5.22E−02	0.740	0.790	< 0.001	***< 0.001	0.043	0.258
Lobule VIIIa (*L*)	3.95E−01 ± 6.30E−02	3.88E−01 ± 5.36E−02	0.660	0.773	0.001	**0.002	0.200	0.504
Lobule VIIIa (vermis)	8.75E−02 ± 1.55E−02	8.92E−02 ± 1.68E−02	0.414	0.609	0.007	**0.009	0.331	0.558
Lobule VIIIa (*R*)	3.53E−01 ± 5.34E−02	3.48E−01 ± 4.67E−02	0.712	0.778	0.009	*0.010	0.234	0.504
Lobule VIIIb (*L*)	3.42E−01 ± 5.20E−02	3.40E−01 ± 4.61E−02	0.999	0.999	0.049	*0.050	0.842	0.850
Lobule VIIIb (vermis)	4.29E−02 ± 7.65E−03	4.44E−02 ± 8.73E−03	0.112	0.301	0.001	**0.002	0.397	0.558
Lobule VIIIb (*R*)	3.34E−01 ± 5.50E−02	3.29E−01 ± 4.57E−02	0.714	0.778	0.221	0.221	0.820	0.850
Lobule IX (*L*)	2.59E−01 ± 4.33E−02	2.61E−01 ± 4.82E−02	0.701	0.778	0.015	*0.018	0.389	0.558
Lobule IX (vermis)	5.66E−02 ± 9.18E−03	5.81E−02 ± 9.38E−03	0.214	0.411	0.004	**0.006	0.164	0.464
Lobule IX (*R*)	2.59E−01 ± 4.30E−02	2.63E−01 ± 4.68E−02	0.451	0.623	0.032	*0.033	0.418	0.558
Lobule X (*L*)	6.37E−02 ± 1.09E−02	6.63E−02 ± 9.48E−03	0.059	0.277	< 0.001	**0.001	0.572	0.638
Lobule X (vermis)	2.71E−02 ± 5.09E−03	2.76E−02 ± 5.07E−03	0.494	0.641	0.009	*0.010	0.559	0.638
Lobule X (*R*)	5.37E−02 ± 9.79E−03	5.49E−02 ± 8.64E−03	0.261	0.463	< 0.001	***< 0.001	0.442	0.558
Dentate nucleus (*L*)	9.67E−02 ± 1.63E−02	1.00E−01 ± 1.53E−02	0.067	0.277	< 0.001	***< 0.001	0.337	0.558
Dentate nucleus (*R*)	1.06E−01 ± 1.84E−02	1.10E−01 ± 1.67E−02	0.113	0.301	< 0.001	***< 0.001	0.253	0.504
Interposed nucleus (*L*)	1.19E−02 ± 2.26E−03	1.22E−02 ± 1.97E−03	0.210	0.411	< 0.001	***< 0.001	0.323	0.558
Interposed nucleus (*R*)	1.23E−02 ± 2.39E−03	1.28E−02 ± 2.43E−03	0.132	0.301	< 0.001	***< 0.001	0.178	0.476
Fastigial nucleus (*L*)	4.05E−03 ± 8.35E−04	4.13E−03 ± 8.28E−04	0.467	0.623	0.028	*0.031	0.700	0.763
Fastigial nucleus (*R*)	3.20E−03 ± 6.86E−04	3.37E−03 ± 7.15E−04	0.047	0.277	< 0.001	***< 0.001	0.262	0.504

Group means (± StDev), raw *p* values, and FDR corrected *p* values from 2 × 2 ANCOVA of basal ganglia and cerebellar volumes, as a percent of total brain volume, covaried for age and MRI scanner. Significant FDR corrected *p* values are bolded and indicated by *p < 0.05; **p < 0.01; ***p < 0.001. All ROIs with significant sex differences were female > male percent of total brain volumes

ROIs were transformed from MNI space to participant space in order to assess volume of each ROI while accounting for total brain volume of the participant. Structural scans from each participant were corrected for signal inhomogeneity and non-linearly registered to MNI space using Advanced Normalization Tools (ANTs) [43]. ROIs were transformed from MNI space to individual participant space using the inverse transformation matrix between the participant and MNI space. For each participant, total volume of each ROI was divided by the total brain volume for that participant, so that ROI volumes were represented as a percent total brain volume. A 2 × 2 ANCOVA was used to assess the effects of diagnosis and sex on percent total brain volume for each of the 48 ROIs, using age and MRI scanner as covariates. We also performed a supplementary analysis for each ROI as absolute volume (mm^3^) using a 2 × 2 ANCOVA with factors of diagnosis and sex, covarying for age, MRI scanner, and total brain volume (in mm^3^). Correction for multiple comparisons was performed using the false discovery rate (FDR) method [44].

#### Free-water and diffusion tensor imaging

Removal of non-CNS tissue was performed with FSL’s Brain Extraction Tool (BET) on the first b0 image and then applied to all remaining diffusion-weighted volumes. Diffusion-weighted scans were corrected for motion and eddy current distortions using affine registration to a reference volume (first b0 image) with the eddy correct function in FSL. Data from FSL’s eddy correct function was then used to rotate the diffusion-weighting directions (i.e., b-vectors), in order to properly estimate the diffusion tensor and diffusion parameters in each voxel after correcting for the distortions caused by motion and eddy currents. Motion was characterized relative to the b0 image for each diffusion-weighted volume. For each diffusion-weighted volume, a summary measure of the total movement across all intracerebral voxels volume was calculated by taking displacement of each voxel and then averaging the squares of those displacements (i.e., root mean square). After applying the initial motion exclusion criteria described under Participant Data (i.e., more than 25% of volumes with > 2 mm motion), we then sought to compare the remaining participant’s motion during diffusion MRI. For each participant, motion across all diffusion-weighted volumes was averaged to arrive at a single motion parameter. We compared motion between ASD and TD groups using a 2 × 2 ANCOVA with the factors of diagnosis and sex, covaried for age and MRI scanner.

**Fig. 1:**
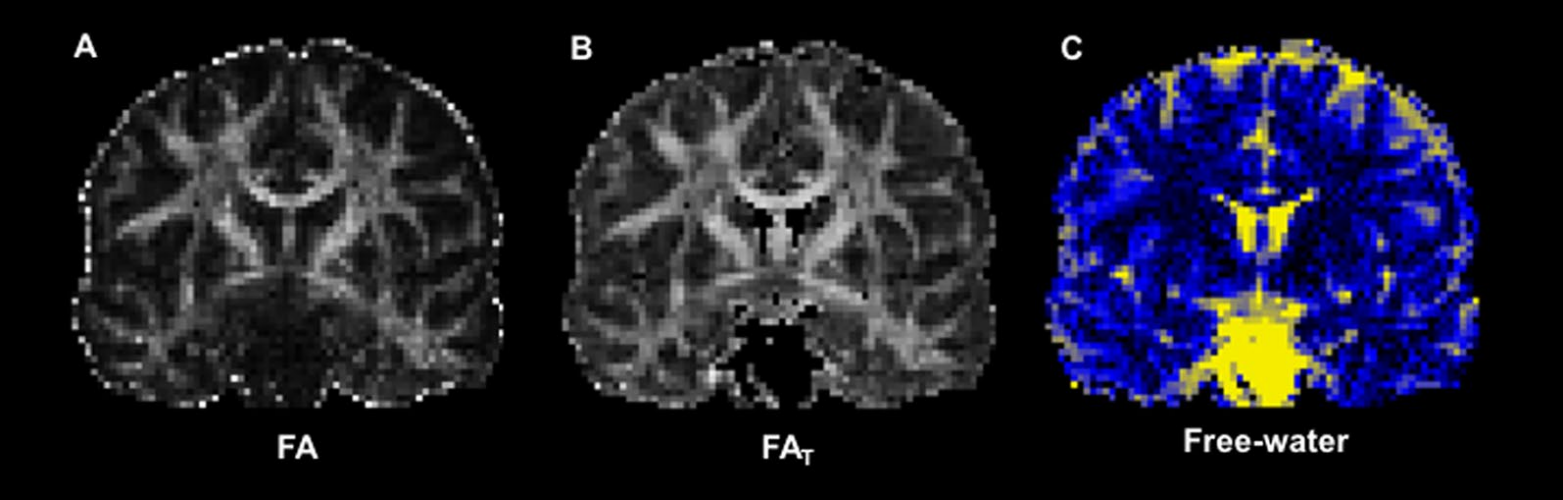
Representative images for diffusion measures. Diffusion imaging data for **A** uncorrected fractional anisotropy (FA), **B** free-water corrected fractional anisotropy (FA_T_), and **C** free-water from a representative TD participant

**Fig. 2:**
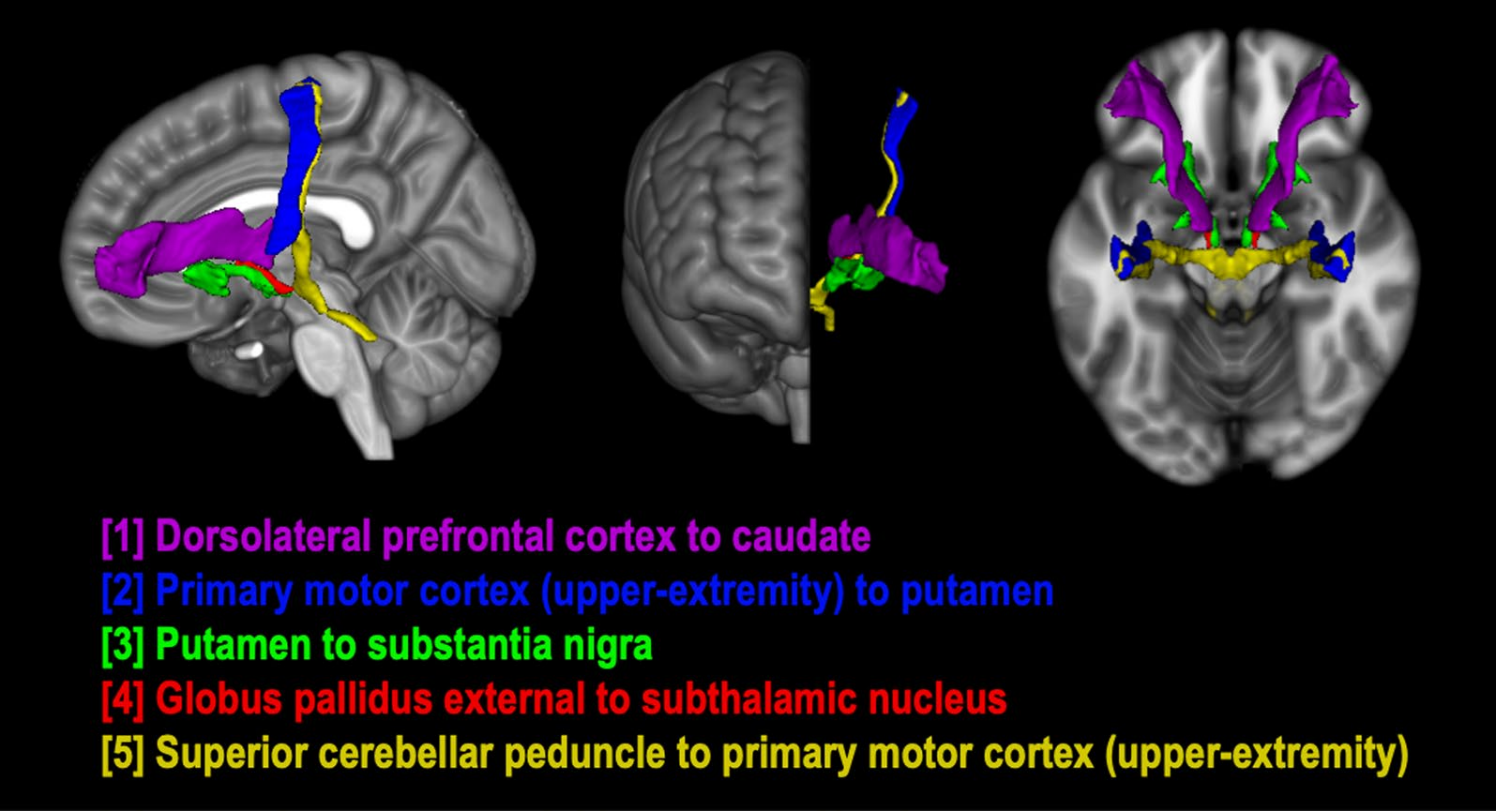
Basal ganglia and cerebellar white matter tracts of interest. Three-dimensional rendering of white matter tract templates depicted from lateral (left), anterior (center), and superior (right) viewpoints. Tracts between the following brain regions were included: (1) Dorsolateral prefrontal cortex to caudate—purple; (2) Primary motor cortex (upper-extremity) to putamen—blue; (3) Substantia nigra to putamen—green; (4) Globus pallidus externa to subthalamic nucleus—red; (5) Superior cerebellar peduncle to primary motor cortex (upper-extremity)—yellow. These tracts were subdivided along the midline into left and right hemispheres for diffusion MRI analyses

We then quantified FW and corrected DTI scalar measures accordingly. FW was calculated using a custom MATLAB script based on Pasternak et al. [13], as described in previous work from our research group [15, 18, 45–49]. We then performed tensor element reconstruction with the DTIFIT function in FMRIB’s Diffusion Toolbox (FDT) to generate free-water corrected fractional anisotropy (FA_T_) maps for each participant. Figure 1 shows uncorrected fractional anisotropy (FA), corrected FA_T_, and FW images from a representative participant in this dataset. We evaluated the following tracts in both left and right hemispheres: (1) Dorsolateral prefrontal cortex (DLPFC) to caudate; (2) Primary motor cortex, upper-extremity (M1-U) to putamen; (3) Substantia nigra (SN) to putamen; (4) Globus pallidus externa (GPe) to subthalamic nucleus (STN); (5) Superior cerebellar peduncle (SCP) to M1-U (see Fig. 2). Templates for the white matter tracts of interest were generated using identical methods to those described in previous work [48, 50, 51]. Briefly, tract templates were generated using probabilistic tractography in the FMRIB software library (FSL; probtrackx2) with slice-level thresholding on high-resolution diffusion imaging data from 100 participants (54 males, 46 females) included in the Human Connectome Project [52]. Seed and target masks were derived from ROIs included the Human Motor Area Template and well-established atlases of the basal ganglia [39, 40, 53, 54]. In the cerebellum, we focused on the SCP tract because prior research showed reduced FA in the SCP of individuals with ASD, which was correlated with motor deficits [55]. FW and FA_T_ were evaluated for both the left and right hemispheres for each of these five tracts, resulting in a total of 10 tracts. We also provide a supplementary analysis of uncorrected FA values in these same tracts.

A 2 × 2 ANCOVA was used to assess the effects of diagnosis (ASD or TD) and sex on FA_T_ and FW for each of the 10 tracts, using age and MRI scanner as covariates. Correction for multiple comparisons was performed with FDR, implemented separately for FA_T_ and FW measures. Next, significant whole-tract differences were followed with slice-level analysis to determine differences in FA_T_ and FW in each slice along the tract. A custom Linux shell-script computed the average FA_T_ and FW at each slice along the primary axis of travel for the tract. These slice-level averages were then compared between ASD and TD groups using independent samples t-tests with FDR correction, similar to previous work [50].

#### Brain-behavior correlations

We performed correlations to assess the association of clinical measures of RRB with basal ganglia and cerebellar volumes, as well as FA_T_ and FW in the white matter tracts. RBS-R scores were available for both the ASD (*n* = 82) and TD (*n* = 93) participants and were correlated with imaging measures using Spearman’s Rho, as the distribution of scores was non-normal. Repetitive behavior scores from section C of the ADI-R, were only available from individuals from the ASD group, and thus correlations for the ADI-R were only preformed within the ASD group. Correction for multiple comparisons was performed with FDR, implemented separately volume, FA_T_, and FW correlations.

## Results

### Demographic data and clinical scales

For IQ scores, there was a significant main effect of diagnosis (*F*_1,183_ = 7.97, *p* = 0.005), no main effect of sex, and no interaction between diagnosis and sex. Post-hoc tests showed that that the ASD group had lower IQ scores than the TD group (Table 1). Regarding participant age, there was no main effect of diagnosis or sex, nor interaction between diagnosis and sex.

For the RBS-R, there was a significant main effect of diagnosis on RBS-R total score (*F*_1,171_ = 114, *p* < 0.001), no main effect of sex, and no interaction between diagnosis and sex. The ASD group had higher RBS-R total scores than the TD group (Table 1). Main effects of diagnosis were also seen for the RBS-R subscales of stereotyped behavior (*F*_1,171_ = 62.3, *p* < 0.001), self-injurious behavior (*F*_1,171_ = 41.1, *p* < 0.001), compulsive behavior (*F*_1,171_ = 42.4, *p* < 0.001), ritualistic behavior (*F*_*1*,171_ = 77.9, *p* < 0.001), sameness behavior (*F*_1,171_ = 77.9, *p* < 0.001). For all subscales, the ASD group had higher scores than the TD group. No main effects of sex, and no interactions between diagnosis and sex were found for these RBS-R subscales.

For the restricted interests subscale there was both a main effect of diagnosis (*F*_1,171_ = 114, *p* < 0.001), a main effect of sex (*F*_1,171_ = 22.1, *p* < 0.001), and an interaction between diagnosis and sex (*F*_1,171_ = 14.6, *p* < 0.001). Post-hoc tests on the restricted interests subscale revealed that males had significantly higher scores than females in both the ASD group (*F*_1,81_ = 17.8, *p* < 0.001) and TD group (*F*_1,90_ = 4.15, *p* = 0.045), although this sex difference was more pronounced in the ASD group.

For the SCQ, there was a main effect of diagnosis (*F*_1,180_ = 394, *p* < 0.001), with the ASD group having significantly higher SCQ scores than the TD group. There was also a main effect of sex (*F*_1,180_ = 10.6, *p* = 0.001), such that males had higher SCQ scores than females. There was no significant interaction between diagnosis and sex.

Scores from section C of the ADI-R were only available for individuals in the ASD group. There was a no effect of sex on ADI-R section C scores.

### Basal ganglia and cerebellar volumes

For ROIs of the basal ganglia and cerebellum, there were no significant main effects of diagnosis, nor interactions between diagnosis and sex on regional gray matter volumes after FDR correction. This was the case whether ROI volume was compared as a percent of total brain volume (Table 2) or as absolute volume (in mm^3^) and covaried by the participant’s total brain volume (Additional file 1: Table S1).

The main effect of sex, which applied equally to both ASD and TD groups, differed between these two approaches for comparing volume. When ROI volume was compared as a percentage of total brain volume, there was a significant main effect of sex (*p*_FDR_ < 0.05) for all ROIs except for right lobule VIIIb, where females had larger percent total brain volume than males in these ROIs (Table 2). When volumes were compared in mm^3^ and covaried by total brain volume (Additional file 1: Table S1), there were fewer ROIs that showed a significant main effect of sex (*p*_FDR_ < 0.05), all of which showed males had larger absolute volume of these structures (due to larger total brain volume male participants): Lobule I–IV (L and R), Lobule V (L and R), Lobule VIIIa (L and R), and Lobule VIIIb (L and R).

### Free-water and diffusion tensor imaging

When comparing head motion between groups, after the initial exclusion criteria for major motion artifacts was applied (i.e., participant excluded if more than 25% of volumes had > 2 mm motion), there was no significant effect of diagnosis (*F*_1,166_ = 1.08, *p* = 0.301), sex (*F*_1,166_ = 0.649, *p* = 0.422), nor interaction between diagnosis and sex (*F*_1,166_ = 0.198, *p* = 0.657) on motion during diffusion MRI.

**Fig. 3:**
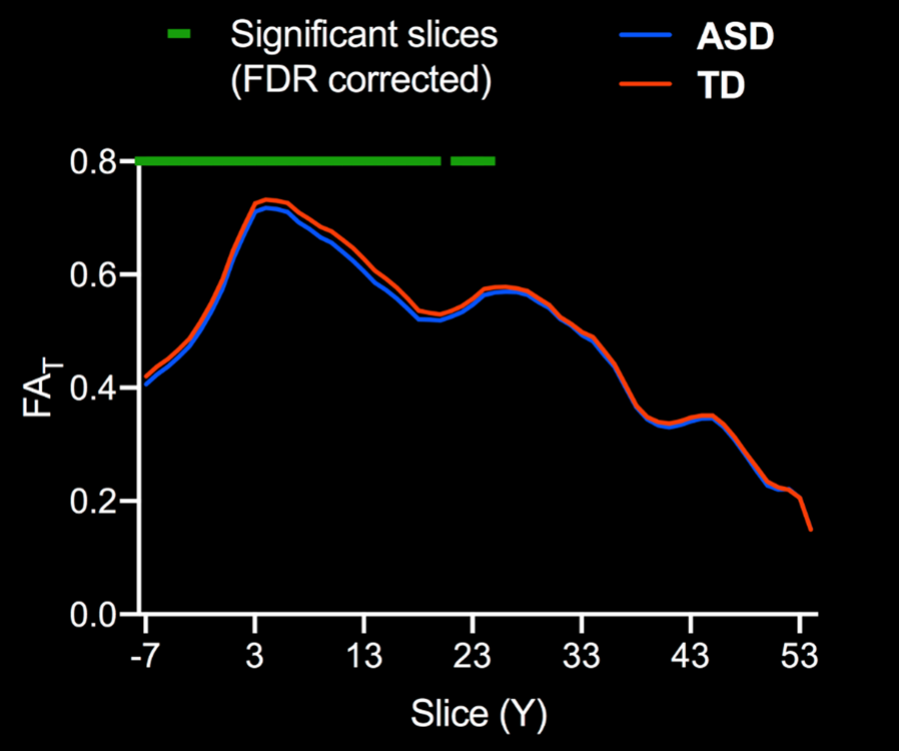
Slice-level analysis of FA_T_ in white matter tracts with significant effect of diagnosis. Slice-level analysis of free-water corrected fractional anisotropy (FA_T_) for the left hemisphere dorsolateral prefrontal cortex to caudate tract (DLPFC to Caudate). Group means for ASD (blue) and TD groups (orange) are depicted. The green line indicates slices with significant differences between groups, FDR corrected for multiple comparisons. Slice numbers in the anterior–posterior (Y) plane are in Talairach coordinates

For FA_T_, there was a significant FDR corrected main effect of diagnosis on the DLPFC to caudate (L) tract (*F*_1,166_ = 11.8, *p*_FDR_ = 0.008), which showed lower FA_T_ in ASD participants. This tract was further investigated using slice-level thresholding, which revealed that slices with significantly lower FA_T_ in ASD participants (*p*_FDR_ < 0.05) were proximal to the caudate (Fig. 3). Although there was no significant interaction between diagnosis and sex for FA_T_ in any of the tracts evaluated, there was a significant FDR corrected main effect of sex for tracts between SN to putamen (L) (*F*_1,166_ = 11.64, *p*_FDR_ = 0.004) and SN to putamen (*R*) (*F*_1,166_ = 20.8, *p*_FDR_ < 0.001), as well as GPe to STN (L) (*F*_1,166_ = 8.50, *p*_FDR_ = 0.014) and GPe to STN (*R*) (*F*_1,166_ = 6.34, *p*_FDR_ = 0.032), all of which showed lower FA_T_ in females than males in both ASD and TD groups. Table 3 summarizes tract average FA_T_ findings.

**Table 3:** Fractional anisotropy (free-water corrected) in basal ganglia and cerebellar white matter tracts

Tract FA_T_	Mean ± Stdev		Diagnosis		Sex		Diagnosis * sex	
	ASD	TD	*p*_raw_	*p*_FDR_	*p*_raw_	*p*_FDR_	*p*_raw_	*p*_FDR_
DLPFC to Caudate (*L*)	0.441 ± 0.014	0.450 ± 0.017	0.001	*0.008	0.069	0.114	0.772	0.956
DLPFC to Caudate (*R*)	0.409 ± 0.017	0.415 ± 0.017	0.014	0.063	0.032	0.063	0.255	0.956
M1U to Putamen (*L*)	0.403 ± 0.016	0.406 ± 0.020	0.290	0.322	0.527	0.585	0.675	0.956
M1U to Putamen (*R*)	0.399 ± 0.017	0.401 ± 0.022	0.367	0.367	0.810	0.810	0.313	0.956
SCP to M1U (*L*)	0.534 ± 0.014	0.537 ± 0.016	0.251	0.314	0.273	0.363	0.694	0.956
SCP to M1U (*R*)	0.520 ± 0.015	0.524 ± 0.019	0.082	0.165	0.290	0.363	0.956	0.956
SN to Putamen (*L*)	0.417 ± 0.016	0.422 ± 0.018	0.108	0.181	0.001	**0.004	0.480	0.956
SN to Putamen (*R*)	0.405 ± 0.017	0.409 ± 0.020	0.152	0.218	< 0.001	***< 0.001	0.371	0.956
GPe to STN (*L*)	0.558 ± 0.019	0.566 ± 0.025	0.045	0.114	0.004	*0.014	0.460	0.956
GPe to STN (*R*)	0.573 ± 0.023	0.584 ± 0.028	0.019	0.063	0.013	*0.032	0.928	0.956

Group means (± StDev), raw *p* values, and FDR corrected *p* values from 2 × 2 ANCOVA for free-water corrected fractional anisotropy (FA_T_) in each white matter tract, covaried for age and MRI scanner. Significant FDR corrected *p* values are bolded and indicated by *p < 0.05; **p < 0.01; ***p < 0.001

The supplementary analysis of uncorrected FA values similarly showed a significant FDR adjusted main effects of diagnosis in the DLPFC to caudate (L) tract (*F*_1,166_ = 21.0, *p*_FDR_ < 0.001) but also in the DLPFC to caudate (*R*) tract (*F*_1,166_ = 12.1, *p*_FDR_ < 0.01). There was a significant main effect of sex for the tracts M1U to caudate (L) (*F*_1,166_ = 17.7, *p*_FDR_ < 0.001) and M1U to caudate (*R*) (*F*_1,166_ = 10.8, *p*_FDR_ < 0.01), as well as GPe to STN (L) (*F*_1,166_ = 11.5, *p*_FDR_ < 0.01) and GPe to STN (*R*) (*F*_1,166_ = 12.1, *p*_FDR_ < 0.01). There was no significant interaction between diagnosis and sex on uncorrected FA in any of the tracts evaluated. Additional file 1: Table S2 summarizes the tract average findings for uncorrected FA.

**Fig. 4:**
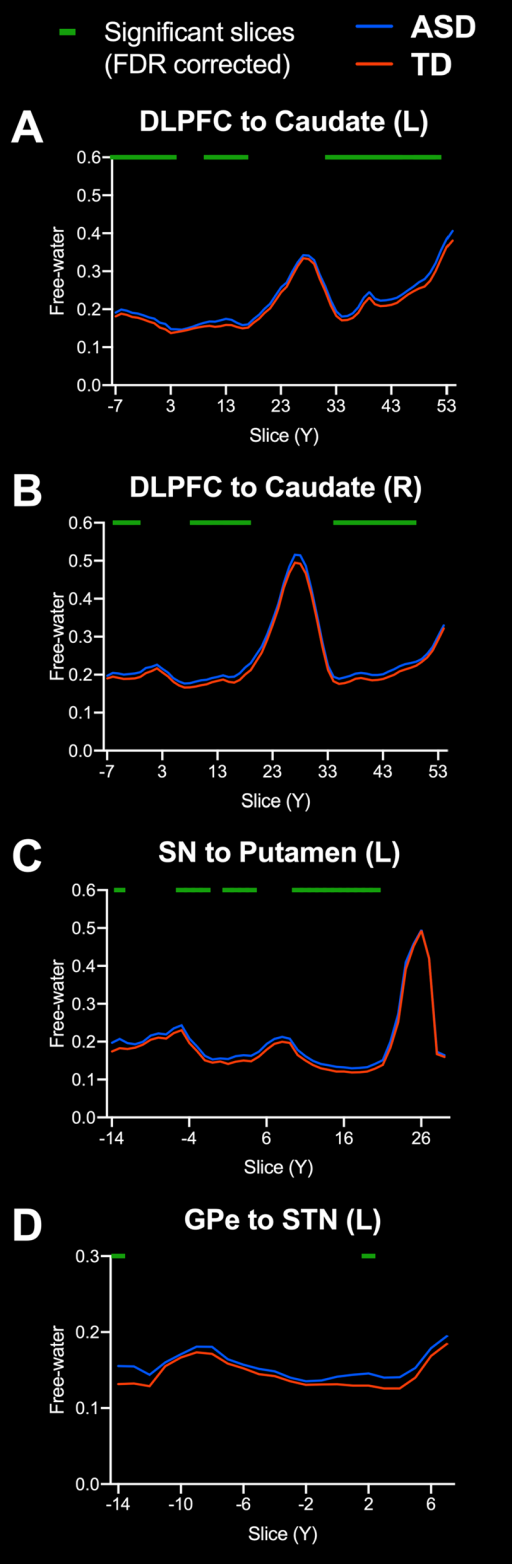
Slice-level analysis of free-water in white matter tracts with a significant effect of diagnosis. Slice-level analysis of free-water (FW) for the **A** left hemisphere dorsolateral prefrontal cortex to caudate (DLPFC to Caudate) tract, **B** right hemisphere DLPFC to caudate tract, **C** left hemisphere substantia nigra to putamen (SN to putamen) tract, and **D** left hemisphere globus pallidus externa to subthalamic nucleus (GPe to STN) tract. Group means for ASD (blue) and TD groups (orange) are depicted. The green line indicates slices with significant differences between groups, FDR corrected for multiple comparisons. Slice numbers in the anterior–posterior (Y) plane are in Talairach coordinates

For FW, there was a significant FDR corrected main effect of diagnosis for DLPFC to caudate (*L*) (*F*_1,166_ = 9.68, *p*_FDR_ = 0.011) and DLPFC to caudate (*R*) (*F*_1,166_ = 5.89, *p*_FDR_ = 0.041), SN to putamen (*L*) (*F*_1,166_ = 10.2, *p*_FDR_ = 0.011), and GPe to STN (*L*) (*F*_1,166_ = 6.90, *p*_FDR_ = 0.031), all of which showed greater FW in ASD participants. These tracts were further investigated using slice-level thresholding. For DLPFC to caudate tracts, slices that had significantly higher FW (*p*_FDR_ < 0.05) were those proximal to both caudate and DLPFC (Fig. 4A, B). For SN to putamen (*L*), slices that had significantly higher FW (*p*_FDR_ < 0.05) were primarily those in the middle of the tract (Fig. 4C). For GPe to STN (*L*), slices that had significantly higher FW (*p*_FDR_ < 0.05) occurred proximal to both GPe and STN (Fig. 4C). There were no significant main effects of sex on FW for any of the tracts investigated, nor were there significant interactions between diagnosis and sex on FW. Table 4 summarizes tract average FW findings.

**Table 4:** Free-water in basal ganglia and cerebellar white matter tracts

Tract FW	Mean ± Stdev		Diagnosis		Sex		Diagnosis * sex	
	ASD	TD	*p*_raw_	*p*_FDR_	*p*_raw_	*p*_FDR_	*p*_raw_	*p*_FDR_
DLPFC to Caudate (*L*)	0.227 ± 0.028	0.214 ± 0.025	0.002	*0.011	0.501	0.974	0.733	0.987
DLPFC to Caudate (*R*)	0.244 ± 0.037	0.230 ± 0.032	0.016	*0.041	0.920	0.974	0.533	0.987
M1U to Putamen (*L*)	0.205 ± 0.041	0.206 ± 0.052	0.611	0.763	0.507	0.974	0.632	0.987
M1U to Putamen (*R*)	0.212 ± 0.040	0.211 ± 0.047	0.915	0.915	0.974	0.974	0.291	0.727
SCP to M1U (*L*)	0.184 ± 0.027	0.185 ± 0.033	0.601	0.763	0.862	0.974	0.987	0.987
SCP to M1U (*R*)	0.197 ± 0.031	0.195 ± 0.030	0.914	0.915	0.724	0.974	0.945	0.987
SN to Putamen (*L*)	0.175 ± 0.024	0.164 ± 0.019	0.002	*0.011	0.633	0.974	0.282	0.727
SN to Putamen (*R*)	0.196 ± 0.029	0.185 ± 0.027	0.049	0.098	0.577	0.974	0.198	0.727
GPe to STN (*L*)	0.156 ± 0.029	0.145 ± 0.022	0.009	*0.031	0.361	0.974	0.224	0.727
GPe to STN (*R*)	0.165 ± 0.030	0.159 ± 0.031	0.344	0.574	0.635	0.974	0.793	0.987

Group means (± StDev), raw *p* values, and FDR corrected *p* values from 2 × 2 ANCOVA for free-water (FW) in each white matter tract, covaried for age and MRI scanner. Significant FDR corrected *p* values are bolded and indicated by *p < 0.05, **p < 0.01, ***p < 0.001

### Brain-behavior correlations

We observed no significant FDR corrected correlations between volume of structures in the basal ganglia and cerebellum with ADI-R Section C scores or SCQ scores. There was a significant negative correlation between RBS-R total score and volume of cerebellar lobule VI (L) (*R* = − 0.25, *p*_FDR_ < 0.05). There were no significant FDR corrected correlations between RBS-R subscale scores and volume for any of the structures measured. Correlations between volume and behavioral measures are depicted in Additional file 1: Table S3.

For FA_T_, there was a significant negative correlation between DLPFC to caudate (*L*) tract FA_T_ and RBS-R total score (*R* = − 0.28, *p*_FDR_ = 0.003), as well as subscales for stereotyped behavior (*R* = − 0.24, *p*_FDR_ = 0.028), ritualistic behavior (*R* = − 0.22, *p*_FDR_ = 0.043), sameness behavior (*R* = − 0.27, *p*_FDR_ = 0.005), and restricted interests (*R* = − 0.27, *p*_FDR_ = 0.005). There was also significant negative correlation between FA_T_ in the DLPFC to caudate (*R*) tract and the RBS-R stereotyped behavior (*R* = − 0.22, *p*_FDR_ = 0.028) and restricted interests subscales (*R* = − 0.22, *p*_FDR_ = 0.026). For the SN to putamen (*L*) tract there were significant negative correlation between FA_T_ and the RBS-R subscale for restricted interests (*R* = − 0.20, *p*_FDR_ = 0.039). For the GPe to STN (*R*) tract there was a significant negative correlation between FA_T_ and the RBS-R subscale for sameness behavior (*R* = − 0.21, *p*_FDR_ = 0.036). Raw and FDR corrected *p* values for correlations between tract FA_T_ and ADI-R section C, RBS-R total score, and RBS-R subscales are included in Table 5.

**Table 5:** Brain-behavior correlations for fractional anisotropy (free-water corrected) in basal ganglia and cerebellar white matter tracts

Tract FA_T_	ADI-R section C		RBS-R total		RBS-R stereotyped		RBS-R self-injury		RBS-R compulsive		RBS-R ritual		RBS-R sameness		RBS-R restricted interests		SCQ	
	*p*_raw_	*p*_FDR_	*p*_raw_	*p*_FDR_	*p*_raw_	*p*_FDR_	*p*_raw_	*p*_FDR_	*p*_raw_	*p*_FDR_	*p*_raw_	*p*_FDR_	*p*_raw_	*p*_FDR_	*p*_raw_	*p*_FDR_	*p*_raw_	*p*_FDR_
DLPFC to Caudate (*L*)	0.500	0.930	< 0.001	**0.003	0.004	*0.028	0.046	0.309	0.016	0.164	0.004	*0.043	< 0.001	**0.005	0.001	**0.005	0.021	0.211
DLPFC to Caudate (*R*)	0.029	0.288	0.025	0.063	0.006	*0.028	0.062	0.309	0.437	0.534	0.189	0.315	0.032	0.089	0.005	*0.026	0.441	0.773
M1U to Putamen (*L*)	0.168	0.839	0.187	0.268	0.367	0.459	0.779	0.918	0.289	0.413	0.770	0.770	0.250	0.312	0.053	0.076	0.696	0.773
M1U to Putamen (*R*)	0.930	0.930	0.565	0.628	0.617	0.617	0.892	0.918	0.164	0.354	0.411	0.587	0.817	0.817	0.829	0.829	0.637	0.773
SCP to M1U (*L*)	0.817	0.930	0.322	0.403	0.513	0.570	0.393	0.786	0.481	0.534	0.649	0.722	0.082	0.117	0.243	0.304	0.799	0.799
SCP to M1U (*R*)	0.661	0.930	0.741	0.741	0.341	0.459	0.918	0.918	0.922	0.922	0.635	0.722	0.329	0.366	0.778	0.829	0.566	0.773
SN to Putamen (*L*)	0.919	0.930	0.020	0.063	0.067	0.130	0.138	0.461	0.118	0.354	0.015	0.066	0.039	0.089	0.012	*0.039	0.208	0.773
SN to Putamen (*R*)	0.334	0.930	0.068	0.114	0.065	0.130	0.228	0.569	0.212	0.354	0.020	0.066	0.049	0.089	0.050	0.076	0.481	0.773
GPe to STN (*L*)	0.894	0.930	0.054	0.109	0.078	0.130	0.899	0.918	0.164	0.354	0.063	0.127	0.053	0.089	0.037	0.076	0.429	0.773
GPe to STN (*R*)	0.727	0.930	0.023	0.063	0.033	0.111	0.770	0.918	0.178	0.354	0.030	0.076	0.007	*0.036	0.041	0.076	0.318	0.773

Raw and FDR corrected *p* values from Spearman correlations between free-water corrected fractional anisotropy (FA_T_) in basal ganglia and cerebellar tracts and behavioral measures. Measures of RRB included Section C of the Autism Diagnostic Interview-Revised (ADI-R), as well Repetitive Behavior Scale-Revised (RBS-R) total score and subsection scores. The Social Communication Questionnaire (SCQ) measures social and communication behaviors. Significant FDR corrected *p *values are bolded and indicated by *p < 0.05, **p < 0.01, ***p < 0.001

The supplementary analysis of correlating uncorrected FA values against behavioral measures showed a significant negative correlation between DLPFC to caudate (*L*) tract uncorrected FA and RBS-R total score (*R* = − 0.35, *p*_FDR_ < 0.001), as well as subscales for stereotyped behavior (*R* = − 0.30, *p*_FDR_ < 0.001), compulsive behavior (*R* = − 0.25, *p*_FDR_ < 0.05), ritualistic behavior (*R* = − 0.32, *p*_FDR_ < 0.001), sameness behavior (*R* = − 0.30, *p*_FDR_ < 0.001), and restricted interests (*R* = − 0.39, *p*_FDR_ < 0.001). There was also a significant negative correlation between DLPFC to caudate (*R*) tract uncorrected FA and RBS-R total score (*R* = − 0.27, *p*_FDR_ < 0.01), as well as subscales for stereotyped behavior (*R* = − 0.29, *p*_FDR_ < 0.001), ritualistic behavior (*R* = − 0.22, *p*_FDR_ < 0.05), sameness behavior (*R* = − 0.25, *p*_FDR_ < 0.01), and restricted interests (*R* = − 0.36, *p*_FDR_ < 0.001). Unlike corrected FA_T_, there was also a significant correlation between uncorrected FA and SCQ scores for the DLPFC to caudate (*L*) (*R* = − 0.28, *p*_FDR_ < 0.01) and DLPFC to caudate (*R*) (*R* = − 0.21, *p*_FDR_ < 0.05) tracts. Correlations between uncorrected FA and behavioral measures are depicted in Additional file 1: Table S4.

For FW, there was a significant positive correlation between DLPFC to caudate (*L*) tract FW with the RBS-R restricted interests subscale (*R* = 0.23, *p*_FDR_ = 0.021) and the SCQ score (*R* = 0.25, *p*_FDR_ = 0.006). For the DLPFC to caudate (*R*) tract FW there were also significant positive correlations with RBS-R restricted interests subscale (*R* = 0.22, *p*_FDR_ = 0.021) and SCQ score (*R* = 0.21, *p*_FDR_ = 0.013). For the SN to putamen (*L*) tract there was a significant positive correlation between FW and SCQ score (*R* = 0.26, *p*_FDR_ = 0.006). For the GPe to STN (L) tract there was a significant positive correlation between FW and SCQ score (*R* = 0.22, *p*_FDR_ = 0.013). Raw and FDR corrected *p *values for correlations between tract FW and ADI-R section C, RBS-R total score, and RBS-R subscales are included in Table 6.

**Table 6:** Brain-behavior correlations for free-water in basal ganglia and cerebellar white matter tracts

Tract free-water	ADI-R section C		RBS-R total		RBS-R stereotyped		RBS-R self-injury		RBS-R compulsive		RBS-R ritual		RBS-R sameness		RBS-R restricted interests		SCQ	
	*p*_raw_	*p*_FDR_	*p*_raw_	*p*_FDR_	*p*_raw_	*p*_FDR_	*p*_raw_	*p*_FDR_	*p*_raw_	*p*_FDR_	*p*_raw_	*p*_FDR_	*p*_raw_	*p*_FDR_	*p*_raw_	*p*_FDR_	*p*_raw_	*p*_FDR_
DLPFC to Caudate (*L*)	0.737	0.886	0.012	0.122	0.204	0.408	0.459	0.995	0.066	0.461	0.010	0.096	0.119	0.694	0.003	*0.021	0.001	**0.006
DLPFC to Caudate (*R*)	0.724	0.886	0.045	0.150	0.096	0.321	0.835	0.995	0.185	0.462	0.045	0.204	0.139	0.694	0.004	*0.021	0.005	*0.013
M1U to Putamen (*L*)	0.886	0.886	0.887	0.907	0.960	0.960	0.963	0.995	0.637	0.909	0.569	0.773	0.888	0.947	0.917	0.917	0.222	0.370
M1U to Putamen (*R*)	0.773	0.886	0.780	0.907	0.690	0.767	0.597	0.995	0.781	0.909	0.841	0.841	0.947	0.947	0.903	0.917	0.386	0.531
SCP to M1U (*L*)	0.208	0.886	0.907	0.907	0.398	0.569	0.581	0.995	0.835	0.909	0.812	0.841	0.664	0.947	0.687	0.858	0.502	0.558
SCP to M1U (*R*)	0.156	0.886	0.815	0.907	0.505	0.631	0.431	0.995	0.424	0.848	0.619	0.773	0.502	0.947	0.580	0.829	0.787	0.787
SN to Putamen (*L*)	0.807	0.886	0.035	0.150	0.082	0.321	0.735	0.995	0.138	0.461	0.061	0.204	0.334	0.834	0.025	0.082	0.001	**0.006
SN to Putamen (*R*)	0.504	0.886	0.289	0.578	0.171	0.408	0.995	0.995	0.618	0.909	0.283	0.567	0.754	0.947	0.128	0.257	0.054	0.108
GPe to STN (*L*)	0.668	0.886	0.070	0.174	0.041	0.321	0.783	0.995	0.127	0.461	0.204	0.510	0.291	0.834	0.105	0.257	0.004	*0.013
GPe to STN (*R*)	0.432	0.886	0.516	0.860	0.396	0.569	0.689	0.995	0.909	0.909	0.592	0.773	0.910	0.947	0.336	0.560	0.425	0.531

Raw and FDR corrected *p* values from Spearman correlations between free-water in basal ganglia and cerebellar tracts and behavioral measures. Measures of RRB included Section C of the Autism Diagnostic Interview-Revised (ADI-R), as well Repetitive Behavior Scale-Revised (RBS-R) total score and subsection scores. The Social Communication Questionnaire (SCQ) measures social and communication behaviors. Significant FDR corrected *p* values are bolded and indicated by *p < 0.05, **p < 0.01, ***p < 0.001

## Discussion

There has been relatively limited focus on morphological and connectivity differences associated with RRB in ASD. An approach frequently employed to assess brain-behavior relationships to RRB in ASD has been correlation of regional gray matter volumes with clinical scales [56–62]. Other work has used fMRI to assess activation or functional connectivity as it relates to RRB [63–71]. Fewer studies, however, have used diffusion imaging to assess white matter correlates of RRB [64, 66, 72–74]. Adding complexity to this line of inquiry, there is mixed evidence regarding sex differences in expression of RRB in ASD [32–36]. Relatively few studies have examined sex differences in ASD using diffusion imaging, and none of these studies investigated the relationship of such findings with RRB [28, 29, 75, 76]. In this study we assessed measures of RRB, regional gray matter volumes and white matter microstructure in the basal ganglia and cerebellum in a large cohort of ASD and TD individuals, with nearly equal numbers of males and females in each group. We also evaluated whether biological sex interacted with ASD diagnosis on these neuroimaging outcome measures, and their relationship to RRB. To our knowledge, this work is the first to assess microstructure of intra-basal ganglia white matter pathways (i.e., SN to putamen, GPe to STN) in ASD and how such pathways relate to expression of RRB. This study is also among the first to quantify FW or implement free-water correction of DTI data in ASD [20, 21].

We observed higher levels of RRB in individuals with ASD, as expected. Overall patterns of RRB were similar between males and females with ASD, as the ADI-R section C and RBS-R total score showed no significant sex differences. There was, however, a significant diagnosis by sex interaction for the restricted interests subscale of the RBS-R. This specific finding regarding restricted interests is in line with other studies that have used the RBS-R to investigate sex differences in the expression of RRB in ASD [34, 36]. We also observed that within TD participants, males showed higher scores on the restricted interests subscale, but this effect was less pronounced than in ASD participants.

Our neuroimaging findings suggest that the gray matter volumes in the basal ganglia and cerebellum, adjusted for total brain volume, do not distinguish children and adolescents with ASD from their TD counterparts. We found no interaction between diagnosis and sex on volume of structures in the basal ganglia and cerebellum, although there were significant sex effects that applied equally to both ASD and TD groups. Previously reported volumetric differences in ASD and their relation to RRB may have been biased by inclusion of only males or very small numbers of females, as well as smaller sample sizes [56–59]. In a larger study of 472 individuals with ASD (54 females), Turner et al. reported that individuals with ASD showed significant volumetric enlargement in the pallidum and lateral ventricles [77]. That study, however, did not investigate potential interactions between diagnosis and sex, but their results were identical when replicated in the male-only group. In a similar study of subcortical volume in 539 individuals with ASD (67 females), Zhang et al. found no effect of diagnostic group for any of the structures evaluated, nor any interaction between diagnosis and sex [30]. It is worth noting that both of those large studies included adults, whereas our study sample included only children and adolescents. There was a significant negative correlation between volume of cerebellar lobule VI (L) and RBS-R total score, an observation which matches Rojas et al. [58], where there was no volumetric difference between ASD and TD individuals in cerebellar lobule VI (L), but a significant negative correlation between repetitive behavior and volume of lobule VI (L) was reported.

In contrast, we found that microstructure of basal ganglia white matter tracts was significantly different in children and adolescents with ASD compared to TD participants. We found that individuals with ASD had lower FA_T_ in the DLPFC to caudate (L) tract, but there was no effect of sex, nor interaction between diagnosis and sex. We also found that individuals with ASD had higher FW in the DLPFC to caudate tracts (L and R), as well as tracts from SN to putamen (L) and GPe to STN (L), with no effect of sex nor interaction between diagnosis and sex. Lower FA_T_ in these tracts, excluding the GPe to STN (L) tract, were correlated with higher scores in at least one subscale of the RBS-R. Higher FW in DLPFC to caudate tracts (L and R) also correlated with higher RBS-R restricted interest scores. Although RBS-R total score and many RBS-R subscales were correlated with white matter microstructure, it is worth noting that no significant correlations were found between self-injury or compulsive behavior subscales with either FA_T_ or FW for any of the tracts examined.

The supplementary analysis of uncorrected FA values showed significant group differences in DLPFC to caudate tracts (L and R). Our objective in including this supplementary analysis is to provide an example of the different conclusions that may be arrived at when applying free-water quantification and correction to diffusion MRI data. In some of the evaluated tracts, such as GPe to STN, uncorrected FA values were nearly 40% greater than corresponding free-water corrected FA_T_ values. The relationship between FA and FA_T_ is determined on a voxel-by-voxel basis and is dependent on the FW fraction [13]. To illustrate the relationship between FA and FA_T_ in the context of this manuscript, we also provide correlations between tract-average FA and FA_T_ in Additional file 1: Table S5. We believe it is especially relevant that the FW metric itself showed greater sensitivity at identifying significant differences between ASD and TD groups, which was observed in both DLPFC to caudate (L and R) tracts, as well as the in basal ganglia tracts SN to putamen (L) and GPe to STN (L).

In order to determine the specificity of these brain-behavior relationships to RRB we also performed correlations with the SCQ, which captures social and communication deficits seen in ASD, but also includes some items pertinent to RRB. There were no significant correlations between SCQ score and regional gray matter volumes in the basal ganglia and cerebellum, nor FA_T_ in the white matter tracts investigated. We found that higher SCQ scores correlated with higher FW in the DLPFC to caudate (L and R), SN to putamen (L), and GPe to STN (L) tracts. The supplementary analysis of uncorrected FA showed that DLPFC to caudate (L and R) was significantly correlated with nearly all RBS-R items as well as the SCQ, but no correlations were observed in other tracts. Considering RBS-R and SCQ findings together, evaluating uncorrected FA may lead to the conclusion that these white matter tracts reflect global severity of both RRB and social deficits. However, following free-water correction, FA_T_ was more specific to RRB and revealed novel involvement of basal ganglia pathways in the expression of RRB.

The DLPFC, which includes middle frontal gyrus and part of superior frontal gyrus [78–80], connects to the basal ganglia via the caudate [81]. Connections from DLPFC to the caudate are thought to play an important role in response inhibition [82]. Aberrant microstructure in the DLPFC to caudate white matter has been shown in other clinical populations with deficits in response inhibition, such as attention-deficit hyperactivity disorder [83] and obsessive–compulsive disorder [84]. Rojas et al. [58] found evidence for altered FA in white matter near the middle frontal gyrus (MFG) in ASD using a voxel-wise approach, but no prior tract-based study has identified aberrant microstructure in white matter connecting the DLPFC to caudate. A previous study in males with ASD found functional connectivity deficits between MFG and caudate, which was further correlated with RRB scores, but found no difference in FA for caudate tracts identified with tractography [64]. Another tractography study in males with ASD found lower FA in putamen tracts, but not in caudate tracts [66].

Quantification of free-water or corresponding correction of DTI scalars has had limited application in ASD research [20, 21]. It is possible that prior evaluations of basal ganglia white matter in ASD using FA as an outcome measure were impacted by partial volume effects of extracellular free-water. This consideration is especially relevant for white matter tracts proximal to ventricles, such as the DLPFC to caudate tract. When we investigated FA_T_ of the DLPFC to caudate tract at slice-level, we found that the slices which had significantly lower FA_T_ in ASD were those most proximal to the ventricles (Fig. 3). The majority of these same DLPFC to caudate slices also had significantly higher FW in ASD (Fig. 4). We did not observe a main effect of sex, nor an interaction between diagnosis and sex, for either FA_T_ or FW in the DLPFC to caudate tract. FA_T_ in the DLPFC to caudate tract was negatively correlated with RBS-R total score and multiple subscales, whereas FW was positively correlated with the restricted interests RBS-R subscale. The supplementary analysis of uncorrected FA also showed significant group differences in ASD for the DLPFC to caudate tracts, and significant correlations for RBS-R and SCQ scores. Together these findings suggest altered microstructure of the DLPFC to caudate tract in ASD, and that higher rates of RRB are associated with greater disruption in this pathway.

The added value of FW imaging can also be seen in white matter pathways within the basal ganglia. Individuals with ASD had higher FW than TD individuals in the SN to putamen tract. Although we did not observe a group difference in FA_T_ for this SN to putamen tract, we found that FA_T_ was negatively correlated with the RBS-R restricted interests subscale. Projections from substantia nigra pars compacta (SNpc) to putamen provide dopaminergic tone to the striatum, and recurrent projections from putamen to SNpc provide regulatory feedback. Also connecting these regions, efferents from the putamen to substantia nigra pars reticulata (SNpr) are part the direct basal ganglia pathway. The SN target mask used in this study did not differentiate between the SNpr and SNpc. Consequently, we did not have the ability to differentiate whether the voxels represented in this tract were comprised of direct pathway GABAergic projections from putamen to SNpr*,* dopaminergic projections from SNpc to putamen, striosomal GABAergic recurrents from putamen to SNpc, or some combination of these.

Due to the multiple distinct white matter populations likely represented in the SN to putamen tract, there are multiple possible interpretations of the present findings for the SN to putamen tract. One possible interpretation is that there is impaired microstructure of the direct pathway in ASD associated with higher levels of RRB. Several theories surrounding RRB postulate an imbalance between direct and indirect pathways [85–87]. Prior studies do not provide evidence of direct pathway dysfunction associated with RRB in ASD, whereas the indirect pathway has been demonstrated to be an important mediator of RRB in work from animal models [87–93]. Another possible interpretation is that the present findings reflect impaired nigrostriatal dopamine tone, due to projections from SNpc to putamen or striosomal recurrent projections from the putamen to SNpc. We find this interpretation more plausible, as nigrostriatal dopamine abnormalities have been hypothesized to underlie RRB in ASD [94]. Moreover, dopamine dysregulation has been linked to RRB in individuals with Parkinson’s disease who receive dopamine replacement therapy, and is thought to relate to impaired activity of the indirect pathway [95, 96].

The GPe to STN tract (L) also had higher FW in individuals with ASD compared to TD participants. FW in the GPe to STN tracts did not correlate with RBS-R scores, but was found to have a positive correlation with SCQ scores. Although we did not observe a significant effect of diagnosis on FA_T_ in GPe to STN tracts, there was a significant negative correlation between FA_T_ in GPe to STN (R) and the RBS-R subscale for sameness behavior, which was further supported by trend level correlations with RBS-R total score and subscale for ritualistic behavior (see Table 6). Connections between the GPe and STN are specific to the indirect basal ganglia pathway, a pathway shown in animal studies to play a critical role in RRB [87–93]. Our findings in this tract support a role for the indirect pathway in ASD, as well as on the severity of RRB and social deficits as indexed by the RBS-R and SCQ, respectively.

A significant main effect of sex on FA_T_ was observed in the putamen to SN tracts (L and R), as well as GPe to STN tracts (L and R). In all of these tracts, males had higher FA_T_ than females. The greater FA_T_ observed in males for these basal ganglia pathways is consistent with a prior DTI investigation of sex differences in TD individuals during childhood and adolescence [97]. We did not observe an effect of sex on FW in any of the evaluated pathways. Importantly, there was no interaction between diagnosis and sex on either FA_T_ or FW for any of the investigated pathways, nor in the clinical scales evaluated in this study. Thus, the main effect of sex was not further explored in this study, as it applied to both ASD and TD populations equally. Nevertheless, other studies suggest that there may be sex differences in brain morphology or connectivity in ASD and it is important that future work in this area continue to evaluate sex as a factor [28, 98, 99].

The lack of significant diffusion imaging findings in cerebellar white matter tracts may be partially related to demographic differences in the study population compared to other similar work. Cheung et al. [73] previously reported a significant negative correlation between RRB and FA in cerebellar white matter. Their investigation included a smaller sample of 13 individuals with ASD, only 1 of which was female, and utilized the ADI-R section C, which yielded no significant correlations in the present study. Regarding age, work has shown that children with ASD show dynamic changes in cerebellar white matter tracts across childhood that differ from their TD counterparts [100]. Moreover, altered FA in cerebellar pathways in infancy and toddlerhood has been associated with the later development of repetitive behaviors and autism. Wolff et al. [101] observed associations between RRB and FA alterations in cerebellar but not basal ganglia white matter tracts, leading them to speculate that these brain regions may play different roles in the development of repetitive behavior over time. The basal ganglia and cerebellum are tightly interconnected, both functionally and structurally, and correspondingly their influence on the development of motor and other behaviors are thought to be intertwined [102]. Thus, the older children in this study may represent a later developmental stage in which repetitive behaviors are more dependent on basal ganglia rather than cerebellar white matter.

Altered FA (or FA_T_) is often considered an indicator of structural connectivity, reflecting factors such as aberrant myelination, but may also correspond to differences in other micro-structural properties (e.g., microtubule density). FW is thought to capture partial volume effects from extracellular space, as the diffusion-weighting time in common imaging sequences is such that FW signal most likely originates from spaces larger than a few tens of microns [103]. In the context of the results from this study, increased FW in ASD may reflect microstructural properties such as reduced density of glial cells in these white matter tracts [104] or neuroinflammation [19]. In support of findings from the present study, research using neurite orientation and dispersion imaging (NODDI) identified higher isotropic volume fraction in white matter of individuals with ASD, which was similarly interpreted as increased extracellular free-water [105]. Brain development in ASD is thought to be affected by “over-pruning” and increased FW could also reflect this process [106]. In conditions such as Parkinson’s disease, FW is also thought to reflect atrophic neurodegeneration [13–16], but we do not suspect neurodegeneration to be driving the present findings from children and adolescents with ASD.

## Limitations

This study was performed on a deidentified dataset from the NDA, and thus we did not control data collection methods. In our data analysis we covaried for scanner type to account for small differences in imaging acquisition parameters, but information regarding the testing site from which each participant’s data originated was unavailable. Thus, this study cannot account for potential variability due to testing site. The white matter pathways evaluated in this study were chosen due to their hypothesized relevance to RRB. Differences between ASD and TD individuals that may occur outside basal ganglia and cerebellar pathways, and their potential relationship to RRB, were not examined. The diffusion MRI data analyzed in this retrospective study were acquired with a single diffusion-weighted shell (*b* = 1000 s/mm^2^). It has been demonstrated that when applying a two-compartment model to single-shell diffusion MRI data, changes in FW may be difficult to disentangle from tissue mean-diffusivity changes, and that multi-shell diffusion data offers improved FW estimates compared to single-shell data [107]. However, when only single-shell data are available, as was the case for this dataset, recent work has shown that applying a two-compartment model (i.e., free-water) provides increased signal-to-noise ratio and greater sensitivity than fitting a single compartment model to single shell data [108]. Although FA_T_ and FW capture aspects of tissue microstructure, the analyses performed here cannot determine the specific microstructural underpinnings of those differences; post-mortem experiments in clinical populations or work in animal models may elucidate how these metrics correspond to microstructural alterations in ASD. Lastly, although mean IQ score in the ASD group was significantly lower than mean IQ of the TD group, individuals in the ASD group had IQ in the normal range. We did not covary for IQ in this study, because it has been suggested that IQ is inappropriate to include as a covariate in studies of neurodevelopmental disorders, as differences in IQ are often part of the phenomena of the condition being studied [109]. It is unknown whether the findings from this study extend to individuals with ASD that have lower IQ scores.

## Conclusions

In this study we used neuroimaging data available from the NDA to assess gray matter volume and white matter microstructure of the basal ganglia and cerebellum in ASD. These novel findings demonstrate that cortico-basal ganglia white matter microstructure is altered in ASD and linked to RRB. FW in cortico-basal ganglia and intra-basal ganglia white matter was more sensitive to group differences in ASD, whereas cortico-basal ganglia FA_T_ was more closely linked to RRB. In contrast, we found no significant difference for basal ganglia or cerebellar gray matter volumes in ASD. Sex-related differences in brain volume and microstructure were present in both ASD and TD groups and did not interact with diagnosis. Prior diffusion imaging investigations of white matter in ASD may have been impacted by partial volume effects of extracellular free-water, especially in basal ganglia pathways proximal to the ventricles. Future diffusion imaging investigations in ASD may benefit from quantification of FW and corresponding correction of FA to account for partial volume effects in order to better understand how basal ganglia white matter is affected in ASD, and how such measures are associated with expression or attenuation of RRB.

### Supplementary Information

**Additional file 1:** Supplementary materials (Tables S1–S5).

## Data Availability

The dataset supporting the conclusions of this article is available in the National Institute of Mental Health Data Archive (NDA): collection ID #2021.

## Abbreviations

ASD: Autism spectrum disorder
RRB: Restricted, repetitive behavior
MRI: Magnetic resonance imaging
DTI: Diffusion tensor imaging
TD: Typically developing
NDA: National Institute of Mental Health Data Archive
ROI(s): Region(s) of interest
IQ: Intelligence quotient
FOV: Field of view
TR: Repetition time
TE: Echo time
ANCOVA: Analysis of covariance
ADI-R: Autism Diagnostic Interview-Revised
RBS-R: Repetitive Behavior Scale-Revised
SCQ: Social Communication Questionnaire
ANTs: Advanced Normalization Tools
BET: Brain Extraction Tool
FDR: False discover rate
FSL: FMRIB Software Library
FA: Fractional anisotropy
FA_T_: Free-water corrected fractional anisotropy
FW: Free-water
DLPFC: Dorsolateral prefrontal cortex
M1-U: Primary motor cortex, upper-extremity
MFG: Middle frontal gyrus
SN: Substantia nigra
SNpc: Substantia nigra pars compacta
SNpr: Substantia nigra pars reticulata
GPe: Globus pallidus externa
STN: Subthalamic nucleus
SCP: Superior cerebellar peduncle
