# Supplementary material for: Cortico-basal ganglia white matter microstructure is linked to restricted repetitive behavior in autism spectrum disorder
Source: Mol Autism. 2024 Jan 23;15:6. doi: 10.1186/s13229-023-00581-2 (PMC10804694; doi:10.1186/s13229-023-00581-2)
Supplement: Supplementary file 1 — Additional file 1: Supplementary materials (Tables S1–S5). [file 13229_2023_581_MOESM1_ESM.docx]

**Table S1: Basal ganglia and cerebellar volumes in mm^3^**

| **REGION OF INTEREST** | **VOLUME IN MM^3^**  **MEAN ± STDEV** | | **DIAGNOSIS** | | | **SEX** | | | **DIAGNOSIS * SEX** | | |  |
| --- | --- | --- | --- | --- | --- | --- | --- | --- | --- | --- | --- | --- |
|  | ASD | TD | p_raw_ | p_FDR_ | | p_raw_ | p_FDR_ | | p_raw_ | p_FDR_ | |  |
| Caudate (L) | 3034 ± 366 | 3026 ± 345 | 0.909 | | 0.976 | 0.218 | | 0.499 | 0.874 | | 1.000 | |
| Caudate (R) | 2847 ± 328 | 2863 ± 319 | 0.755 | | 0.954 | 0.275 | | 0.549 | 0.641 | | 1.000 | |
| Putamen (L) | 5048 ± 452 | 5149 ± 388 | 0.080 | | 0.397 | 0.844 | | 0.940 | 0.181 | | 0.921 | |
| Putamen (R) | 4896 ± 464 | 5000 ± 388 | 0.064 | | 0.397 | 0.872 | | 0.940 | 0.136 | | 0.921 | |
| Nucleus accumbens (L) | 1130 ± 109 | 1143 ± 111 | 0.294 | | 0.578 | 0.394 | | 0.631 | 0.712 | | 1.000 | |
| Nucleus accumbens (R) | 1088 ± 104 | 1112 ± 94 | 0.056 | | 0.397 | 0.024 | | 0.107 | 0.673 | | 1.000 | |
| Globus pallidus external (L) | 922 ± 98 | 934 ± 80 | 0.598 | | 0.869 | 0.340 | | 0.595 | 0.069 | | 0.921 | |
| Globus pallidus external (R) | 848 ± 91 | 864 ± 75 | 0.370 | | 0.612 | 0.763 | | 0.886 | 0.249 | | 0.921 | |
| Globus pallidus internal (L) | 334 ± 38 | 339 ± 36 | 0.688 | | 0.898 | 0.911 | | 0.951 | 0.028 | | 0.733 | |
| Globus pallidus internal (R) | 313 ± 37 | 326 ± 37 | 0.035 | | 0.397 | 0.312 | | 0.595 | 0.216 | | 0.921 | |
| Subthalamic nucleus (L) | 75 ± 11 | 75 ± 12 | 0.870 | | 0.976 | 0.097 | | 0.273 | 0.532 | | 1.000 | |
| Subthalamic nucleus (R) | 74 ± 10 | 73 ± 11 | 0.189 | | 0.493 | 0.771 | | 0.886 | 0.154 | | 0.921 | |
| Substantia nigra (L) | 169 ± 18 | 172 ± 22 | 0.648 | | 0.889 | 0.205 | | 0.492 | 0.698 | | 1.000 | |
| Substantia nigra (R) | 171 ± 18 | 175 ± 21 | 0.301 | | 0.578 | 0.660 | | 0.856 | 0.207 | | 0.921 | |
| Lobule I-IV (L) | 3963 ± 484 | 3978 ± 494 | 0.830 | | 0.976 | 0.003 | | ***0.022** | 0.879 | | 1.000 | |
| Lobule I-IV (R) | 4117 ± 494 | 4154 ± 443 | 0.920 | | 0.976 | <0.001 | | ****0.006** | 0.999 | | 1.000 | |
| Lobule V (L) | 4675 ± 470 | 4784 ± 503 | 0.248 | | 0.567 | 0.006 | | ***0.035** | 0.378 | | 1.000 | |
| Lobule V (R) | 4777 ± 511 | 4907 ± 516 | 0.183 | | 0.493 | 0.001 | | ***0.012** | 0.455 | | 1.000 | |
| Lobule VI (L) | 8905 ± 1157 | 9313 ± 926 | 0.027 | | 0.397 | 0.021 | | 0.100 | 0.354 | | 1.000 | |
| Lobule VI (vermis) | 2137 ± 291 | 2190 ± 320 | 0.438 | | 0.700 | 0.089 | | 0.267 | 0.941 | | 1.000 | |
| Lobule VI (R) | 7917 ± 922 | 8264 ± 926 | 0.025 | | 0.397 | 0.496 | | 0.721 | 0.389 | | 1.000 | |
| Crus I (L) | 14512 ± 1443 | 14856 ± 1550 | 0.151 | | 0.453 | 0.592 | | 0.836 | 0.031 | | 0.733 | |
| Crus I (vermis) | 16 ± 4 | 16 ± 4 | 0.912 | | 0.976 | 0.371 | | 0.615 | 0.467 | | 1.000 | |
| Crus I (R) | 14560 ± 1323 | 14964 ± 1459 | 0.077 | | 0.397 | 0.424 | | 0.636 | 0.877 | | 1.000 | |
| Crus II (L) | 10844 ± 1024 | 10830 ± 1340 | 0.856 | | 0.976 | 0.775 | | 0.886 | 0.701 | | 1.000 | |
| Crus II (vermis) | 514 ± 78 | 535 ± 86 | 0.125 | | 0.428 | 0.421 | | 0.636 | 0.595 | | 1.000 | |
| Crus II (R) | 10404 ± 986 | 10543 ± 1084 | 0.342 | | 0.609 | 1.000 | | 1.000 | 0.928 | | 1.000 | |
| Lobule VIIb (L) | 5074 ± 511 | 4971 ± 478 | 0.099 | | 0.397 | 0.069 | | 0.255 | 0.959 | | 1.000 | |
| Lobule VIIb (vermis) | 182 ± 31 | 189 ± 32 | 0.195 | | 0.493 | 0.333 | | 0.595 | 0.572 | | 1.000 | |
| Lobule VIIb (R) | 5417 ± 487 | 5355 ± 470 | 0.361 | | 0.612 | 0.347 | | 0.595 | 0.196 | | 0.921 | |
| Lobule VIIIa (L) | 5603 ± 595 | 5472 ± 520 | 0.043 | | 0.397 | 0.003 | | ***0.022** | 0.964 | | 1.000 | |
| Lobule VIIIa (vermis) | 1243 ± 163 | 1258 ± 192 | 0.869 | | 0.976 | 0.232 | | 0.505 | 0.955 | | 1.000 | |
| Lobule VIIIa (R) | 5012 ± 430 | 4915 ± 464 | 0.053 | | 0.397 | <0.001 | | ****0.003** | 0.864 | | 1.000 | |
| Lobule VIIIb (L) | 4853 ± 523 | 4796 ± 503 | 0.271 | | 0.578 | <0.001 | | ****0.003** | 0.185 | | 0.921 | |
| Lobule VIIIb (vermis) | 610 ± 84 | 627 ± 102 | 0.248 | | 0.567 | 0.774 | | 0.886 | 0.874 | | 1.000 | |
| Lobule VIIIb (R) | 4733 ± 520 | 4646 ± 505 | 0.099 | | 0.397 | <0.001 | | *****<0.001** | 0.177 | | 0.921 | |
| Lobule IX (L) | 3687 ± 502 | 3680 ± 563 | 0.692 | | 0.898 | 0.129 | | 0.343 | 0.885 | | 1.000 | |
| Lobule IX (vermis) | 805 ± 102 | 820 ± 108 | 0.486 | | 0.728 | 0.200 | | 0.492 | 0.639 | | 1.000 | |
| Lobule IX (R) | 3691 ± 515 | 3719 ± 564 | 0.935 | | 0.976 | 0.085 | | 0.267 | 0.951 | | 1.000 | |
| Lobule X (L) | 903 ± 101 | 934 ± 90 | 0.094 | | 0.397 | 0.021 | | 0.100 | 0.243 | | 0.921 | |
| Lobule X (vermis) | 384 ± 57 | 390 ± 57 | 0.993 | | 0.993 | 0.084 | | 0.267 | 0.541 | | 1.000 | |
| Lobule X (R) | 760 ± 90 | 774 ± 84 | 0.634 | | 0.889 | 0.244 | | 0.509 | 0.405 | | 1.000 | |
| Dentate nucleus (L) | 1370 ± 152 | 1411 ± 145 | 0.140 | | 0.449 | 0.631 | | 0.841 | 0.750 | | 1.000 | |
| Dentate nucleus (R) | 1508 ± 179 | 1545 ± 152 | 0.281 | | 0.578 | 0.998 | | 1.000 | 0.835 | | 1.000 | |
| Interposed nucleus (L) | 169 ± 24 | 173 ± 20 | 0.470 | | 0.728 | 0.764 | | 0.886 | 1.000 | | 1.000 | |
| Interposed nucleus (R) | 175 ± 25 | 180 ± 26 | 0.315 | | 0.581 | 0.628 | | 0.841 | 0.830 | | 1.000 | |
| Fastigial nucleus (L) | 57 ± 10 | 58 ± 9 | 0.992 | | 0.993 | 0.064 | | 0.255 | 0.451 | | 1.000 | |
| Fastigial nucleus (R) | 45 ± 8 | 47 ± 8 | 0.116 | | 0.428 | 0.881 | | 0.940 | 0.971 | | 1.000 | |

**Table S1 legend:** Group means (±StDev), raw p-values, and FDR corrected p-values from 2x2 ANCOVA of basal ganglia and cerebellar volumes (in mm^3^), covaried for age, MRI scanner, and total brain volume. Significant FDR corrected p-values are bolded and indicated by *****p<0.05, ******p<0.01, *******p<0.001. All ROIs with significant sex differences using this absolute volume (mm^3^) approach were male > female .

**Table S2:** Fractional anisotropy (uncorrected) in basal ganglia and cerebellar white matter tracts.

| **TRACT FA** | **MEAN ± STDEV** | | | **DIAGNOSIS** | | | **SEX** | | | **DIAGNOSIS * SEX** | | | |  |
| --- | --- | --- | --- | --- | --- | --- | --- | --- | --- | --- | --- | --- | --- | --- |
|  | ASD | TD | | p_raw_ | p_FDR_ | | p_raw_ | p_FDR_ | | | p_raw_ | p_FDR_ | |  |
| DLPFC to Caudate (L) | 0.354 ± 0.020 | | 0.371 ± 0.026 | <0.001 | | *****<0.001** | 0.236 | | 0.393 | 0.777 | | | 0.970 | |
| DLPFC to Caudate (R) | 0.307 ± 0.019 | | 0.320 ± 0.026 | <0.001 | | ****0.003** | 0.145 | | 0.290 | 0.837 | | | 0.970 | |
| M1U to Putamen (L) | 0.504 ± 0.036 | | 0.515 ± 0.050 | 0.217 | | 0.217 | <0.001 | | *****<0.001** | 0.950 | | | 0.970 | |
| M1U to Putamen (R) | 0.507 ± 0.041 | | 0.520 ± 0.050 | 0.161 | | 0.179 | 0.001 | | ****0.003** | 0.668 | | | 0.970 | |
| SCP to M1U (L) | 0.345 ± 0.023 | | 0.354 ± 0.031 | 0.065 | | 0.145 | 0.299 | | 0.405 | 0.924 | | | 0.970 | |
| SCP to M1U (R) | 0.344 ± 0.021 | | 0.352 ± 0.032 | 0.071 | | 0.145 | 0.646 | | 0.646 | 0.699 | | | 0.970 | |
| SN to Putamen (L) | 0.460 ± 0.020 | | 0.467 ± 0.030 | 0.073 | | 0.145 | 0.376 | | 0.417 | 0.231 | | | 0.970 | |
| SN to Putamen (R) | 0.442 ± 0.023 | | 0.448 ± 0.032 | 0.123 | | 0.154 | 0.324 | | 0.405 | 0.564 | | | 0.970 | |
| GPe to STN (L) | 0.343 ± 0.022 | | 0.352 ± 0.029 | 0.087 | | 0.145 | <0.001 | | ****0.003** | 0.970 | | | 0.970 | |
| GPe to STN (R) | 0.330 ± 0.022 | | 0.338 ± 0.032 | 0.118 | | 0.154 | <0.001 | | ****0.003** | 0.438 | | | 0.970 | |

**Table S2 legend:** Group means (±StDev), raw p-values, and FDR corrected p-values from 2x2 ANCOVA for uncorrected fractional anisotropy (FA) in each white matter tract, covaried for age and MRI scanner. Significant FDR corrected p-values are bolded and indicated by *****p<0.05, ******p<0.01, *******p<0.001.

**Table S3:** Spearman correlations between regional brain volume (percent total brain volume) and measures of RRB.

| **REGION OF INTEREST** | **ADI-R Section C** | | **RBS-R Total** | | **RBS-R Stereotyped** | | **RBS-R**  **Self-Injury** | | **RBS-R Compulsive** | | **RBS-R Ritual** | | **RBS-R Sameness** | | **RBS-R Restricted Interests** | | **SCQ** | |
| --- | --- | --- | --- | --- | --- | --- | --- | --- | --- | --- | --- | --- | --- | --- | --- | --- | --- | --- |
|  | p_raw_ | p_FDR_ | p_raw_ | p_FDR_ | p_raw_ | p_FDR_ | p_raw_ | p_FDR_ | p_raw_ | p_FDR_ | p_raw_ | p_FDR_ | p_raw_ | p_FDR_ | p_raw_ | p_FDR_ | p_raw_ | p_FDR_ |
| Caudate (L) | 0.134 | 0.891 | 0.672 | 0.717 | 0.322 | 0.804 | 0.803 | 0.997 | 0.375 | 0.599 | 0.745 | 0.795 | 0.929 | 0.949 | 0.741 | 0.825 | 0.706 | 0.949 |
| Caudate (R) | 0.222 | 0.891 | 0.479 | 0.535 | 0.479 | 0.862 | 0.800 | 0.997 | 0.150 | 0.425 | 0.436 | 0.537 | 0.887 | 0.925 | 0.688 | 0.786 | 0.490 | 0.945 |
| Putamen (L) | 0.458 | 0.891 | 0.165 | 0.264 | 0.850 | 0.977 | 0.890 | 0.997 | 0.423 | 0.599 | 0.211 | 0.316 | 0.431 | 0.686 | 0.090 | 0.301 | 0.571 | 0.945 |
| Putamen (R) | 0.284 | 0.891 | 0.191 | 0.296 | 0.740 | 0.933 | 0.778 | 0.997 | 0.454 | 0.599 | 0.235 | 0.331 | 0.433 | 0.686 | 0.137 | 0.348 | 0.677 | 0.949 |
| Nucleus accumbens (L) | 0.817 | 0.933 | 0.427 | 0.512 | 0.978 | 0.980 | 0.467 | 0.997 | 0.522 | 0.626 | 0.297 | 0.396 | 0.805 | 0.916 | 0.255 | 0.489 | 0.369 | 0.945 |
| Nucleus accumbens (R) | 0.816 | 0.933 | 0.095 | 0.219 | 0.523 | 0.877 | 0.861 | 0.997 | 0.372 | 0.599 | 0.105 | 0.249 | 0.357 | 0.686 | 0.107 | 0.301 | 0.254 | 0.945 |
| Globus pallidus external (L) | 0.546 | 0.891 | 0.299 | 0.422 | 0.937 | 0.977 | 0.362 | 0.997 | 0.474 | 0.599 | 0.103 | 0.249 | 0.662 | 0.905 | 0.212 | 0.442 | 0.553 | 0.945 |
| Globus pallidus external (R) | 0.712 | 0.933 | 0.119 | 0.219 | 0.741 | 0.933 | 0.871 | 0.997 | 0.400 | 0.599 | 0.049 | 0.214 | 0.437 | 0.686 | 0.099 | 0.301 | 0.401 | 0.945 |
| Globus pallidus internal (L) | 0.532 | 0.891 | 0.293 | 0.422 | 0.874 | 0.977 | 0.788 | 0.997 | 0.623 | 0.712 | 0.198 | 0.316 | 0.589 | 0.832 | 0.268 | 0.495 | 0.658 | 0.949 |
| Globus pallidus internal (R) | 0.692 | 0.933 | 0.044 | 0.142 | 0.233 | 0.800 | 0.832 | 0.997 | 0.513 | 0.626 | 0.055 | 0.220 | 0.142 | 0.523 | 0.045 | 0.267 | 0.399 | 0.945 |
| Subthalamic nucleus (L) | 0.841 | 0.938 | 0.372 | 0.483 | 0.548 | 0.877 | 0.911 | 0.997 | 0.798 | 0.851 | 0.599 | 0.685 | 0.690 | 0.905 | 0.239 | 0.479 | 0.377 | 0.945 |
| Subthalamic nucleus (R) | 0.576 | 0.891 | 0.420 | 0.512 | 0.363 | 0.804 | 0.914 | 0.997 | 0.842 | 0.878 | 0.592 | 0.685 | 0.443 | 0.686 | 0.493 | 0.695 | 0.816 | 0.959 |
| Substantia nigra (L) | 0.888 | 0.947 | 0.034 | 0.127 | 0.275 | 0.804 | 0.952 | 0.997 | 0.251 | 0.490 | 0.154 | 0.283 | 0.036 | 0.392 | 0.071 | 0.301 | 0.731 | 0.949 |
| Substantia nigra (R) | 0.996 | 0.996 | 0.011 | 0.105 | 0.054 | 0.486 | 0.440 | 0.997 | 0.293 | 0.541 | 0.025 | 0.171 | 0.008 | 0.365 | 0.031 | 0.267 | 0.549 | 0.945 |
| Lobule I-IV (L) | 0.425 | 0.891 | 0.354 | 0.472 | 0.980 | 0.980 | 0.983 | 0.997 | 0.331 | 0.568 | 0.525 | 0.630 | 0.829 | 0.916 | 0.773 | 0.825 | 0.599 | 0.949 |
| Lobule I-IV (R) | 0.870 | 0.947 | 0.096 | 0.219 | 0.577 | 0.884 | 0.676 | 0.997 | 0.122 | 0.425 | 0.165 | 0.283 | 0.423 | 0.686 | 0.415 | 0.622 | 0.922 | 0.984 |
| Lobule V (L) | 0.738 | 0.933 | 0.123 | 0.219 | 0.655 | 0.924 | 0.847 | 0.997 | 0.255 | 0.490 | 0.206 | 0.316 | 0.425 | 0.686 | 0.314 | 0.537 | 0.822 | 0.959 |
| Lobule V (R) | 0.266 | 0.891 | 0.018 | 0.106 | 0.060 | 0.486 | 0.727 | 0.997 | 0.016 | 0.261 | 0.082 | 0.249 | 0.074 | 0.460 | 0.138 | 0.348 | 0.501 | 0.945 |
| Lobule VI (L) | 0.130 | 0.891 | 0.001 | ***0.047** | 0.017 | 0.486 | 0.252 | 0.997 | 0.001 | 0.057 | 0.002 | 0.108 | 0.017 | 0.392 | 0.030 | 0.267 | 0.017 | 0.789 |
| Lobule VI (vermis) | 0.163 | 0.891 | 0.123 | 0.219 | 0.185 | 0.683 | 0.997 | 0.997 | 0.425 | 0.599 | 0.156 | 0.283 | 0.361 | 0.686 | 0.309 | 0.537 | 0.567 | 0.945 |
| Lobule VI (R) | 0.318 | 0.891 | 0.004 | 0.089 | 0.066 | 0.486 | 0.451 | 0.997 | 0.012 | 0.261 | 0.006 | 0.149 | 0.041 | 0.392 | 0.102 | 0.301 | 0.042 | 0.789 |
| Crus I (L) | 0.059 | 0.891 | 0.032 | 0.127 | 0.121 | 0.646 | 0.924 | 0.997 | 0.102 | 0.425 | 0.023 | 0.171 | 0.131 | 0.523 | 0.064 | 0.301 | 0.057 | 0.789 |
| Crus I (vermis) | 0.284 | 0.891 | 0.515 | 0.562 | 0.485 | 0.862 | 0.753 | 0.997 | 0.885 | 0.896 | 0.628 | 0.702 | 0.990 | 0.990 | 0.550 | 0.695 | 0.989 | 0.996 |
| Crus I (R) | 0.185 | 0.891 | 0.043 | 0.142 | 0.278 | 0.804 | 0.970 | 0.997 | 0.114 | 0.425 | 0.011 | 0.149 | 0.196 | 0.627 | 0.202 | 0.441 | 0.072 | 0.789 |
| Crus II (L) | 0.662 | 0.933 | 0.308 | 0.422 | 0.917 | 0.977 | 0.369 | 0.997 | 0.403 | 0.599 | 0.436 | 0.537 | 0.697 | 0.905 | 0.541 | 0.695 | 0.909 | 0.984 |
| Crus II (vermis) | 0.146 | 0.891 | 0.022 | 0.119 | 0.070 | 0.486 | 0.535 | 0.997 | 0.184 | 0.425 | 0.087 | 0.249 | 0.054 | 0.435 | 0.051 | 0.274 | 0.283 | 0.945 |
| Crus II (R) | 0.758 | 0.933 | 0.063 | 0.177 | 0.369 | 0.804 | 0.406 | 0.997 | 0.179 | 0.425 | 0.085 | 0.249 | 0.226 | 0.679 | 0.092 | 0.301 | 0.388 | 0.945 |
| Lobule VIIb (L) | 0.784 | 0.933 | 0.806 | 0.806 | 0.457 | 0.862 | 0.205 | 0.997 | 0.896 | 0.896 | 0.875 | 0.881 | 0.439 | 0.686 | 0.763 | 0.825 | 0.337 | 0.945 |
| Lobule VIIb (vermis) | 0.278 | 0.891 | 0.015 | 0.106 | 0.071 | 0.486 | 0.635 | 0.997 | 0.183 | 0.425 | 0.044 | 0.210 | 0.086 | 0.460 | 0.085 | 0.301 | 0.628 | 0.949 |
| Lobule VIIb (R) | 0.800 | 0.933 | 0.450 | 0.516 | 0.921 | 0.977 | 0.573 | 0.997 | 0.622 | 0.712 | 0.801 | 0.836 | 0.796 | 0.916 | 0.844 | 0.881 | 0.859 | 0.959 |
| Lobule VIIIa (L) | 0.284 | 0.891 | 0.775 | 0.791 | 0.903 | 0.977 | 0.234 | 0.997 | 0.468 | 0.599 | 0.670 | 0.731 | 0.762 | 0.916 | 0.901 | 0.920 | 0.498 | 0.945 |
| Lobule VIIIa (vermis) | 0.539 | 0.891 | 0.121 | 0.219 | 0.336 | 0.804 | 0.631 | 0.997 | 0.136 | 0.425 | 0.165 | 0.283 | 0.555 | 0.831 | 0.342 | 0.567 | 0.948 | 0.989 |
| Lobule VIIIa (R) | 0.501 | 0.891 | 0.726 | 0.757 | 0.596 | 0.884 | 0.381 | 0.997 | 0.315 | 0.560 | 0.881 | 0.881 | 0.854 | 0.916 | 0.620 | 0.744 | 0.429 | 0.945 |
| Lobule VIIIb (L) | 0.028 | 0.891 | 0.138 | 0.229 | 0.608 | 0.884 | 0.731 | 0.997 | 0.062 | 0.425 | 0.134 | 0.280 | 0.282 | 0.686 | 0.567 | 0.698 | 0.797 | 0.959 |
| Lobule VIIIb (vermis) | 0.297 | 0.891 | 0.082 | 0.207 | 0.183 | 0.683 | 0.936 | 0.997 | 0.242 | 0.490 | 0.097 | 0.249 | 0.300 | 0.686 | 0.039 | 0.267 | 0.554 | 0.945 |
| Lobule VIIIb (R) | 0.115 | 0.891 | 0.403 | 0.509 | 0.757 | 0.933 | 0.681 | 0.997 | 0.131 | 0.425 | 0.373 | 0.484 | 0.771 | 0.916 | 0.967 | 0.967 | 0.338 | 0.945 |
| Lobule IX (L) | 0.213 | 0.891 | 0.137 | 0.229 | 0.471 | 0.862 | 0.382 | 0.997 | 0.116 | 0.425 | 0.093 | 0.249 | 0.338 | 0.686 | 0.534 | 0.695 | 0.996 | 0.996 |
| Lobule IX (vermis) | 0.414 | 0.891 | 0.081 | 0.207 | 0.330 | 0.804 | 0.686 | 0.997 | 0.157 | 0.425 | 0.156 | 0.283 | 0.246 | 0.686 | 0.389 | 0.622 | 0.775 | 0.959 |
| Lobule IX (R) | 0.436 | 0.891 | 0.032 | 0.127 | 0.301 | 0.804 | 0.117 | 0.997 | 0.038 | 0.425 | 0.041 | 0.210 | 0.125 | 0.523 | 0.410 | 0.622 | 0.724 | 0.949 |
| Lobule X (L) | 0.558 | 0.891 | 0.006 | 0.089 | 0.063 | 0.486 | 0.582 | 0.997 | 0.235 | 0.490 | 0.012 | 0.149 | 0.034 | 0.392 | 0.040 | 0.267 | 0.242 | 0.945 |
| Lobule X (vermis) | 0.764 | 0.933 | 0.284 | 0.422 | 0.758 | 0.933 | 0.552 | 0.997 | 0.076 | 0.425 | 0.218 | 0.316 | 0.798 | 0.916 | 0.679 | 0.786 | 0.841 | 0.959 |
| Lobule X (R) | 0.725 | 0.933 | 0.121 | 0.219 | 0.675 | 0.926 | 0.878 | 0.997 | 0.448 | 0.599 | 0.043 | 0.210 | 0.407 | 0.686 | 0.429 | 0.624 | 0.280 | 0.945 |
| Dentate nucleus (L) | 0.363 | 0.891 | 0.007 | 0.089 | 0.142 | 0.682 | 0.421 | 0.997 | 0.186 | 0.425 | 0.025 | 0.171 | 0.080 | 0.460 | 0.008 | 0.192 | 0.130 | 0.945 |
| Dentate nucleus (R) | 0.389 | 0.891 | 0.018 | 0.106 | 0.182 | 0.683 | 0.634 | 0.997 | 0.150 | 0.425 | 0.061 | 0.226 | 0.165 | 0.567 | 0.026 | 0.267 | 0.185 | 0.945 |
| Interposed nucleus (L) | 0.538 | 0.891 | 0.115 | 0.219 | 0.426 | 0.862 | 0.783 | 0.997 | 0.770 | 0.840 | 0.266 | 0.364 | 0.443 | 0.686 | 0.150 | 0.355 | 0.293 | 0.945 |
| Interposed nucleus (R) | 0.939 | 0.963 | 0.027 | 0.127 | 0.086 | 0.515 | 0.901 | 0.997 | 0.173 | 0.425 | 0.109 | 0.249 | 0.111 | 0.523 | 0.005 | 0.192 | 0.349 | 0.945 |
| Fastigial nucleus (L) | 0.520 | 0.891 | 0.451 | 0.516 | 0.810 | 0.972 | 0.700 | 0.997 | 0.669 | 0.747 | 0.206 | 0.316 | 0.859 | 0.916 | 0.527 | 0.695 | 0.665 | 0.949 |
| Fastigial nucleus (R) | 0.943 | 0.963 | 0.056 | 0.167 | 0.539 | 0.877 | 0.504 | 0.997 | 0.130 | 0.425 | 0.128 | 0.280 | 0.571 | 0.831 | 0.155 | 0.355 | 0.082 | 0.789 |

**Table S3 legend:** Raw and 5% FDR corrected p-values from Spearman correlations between regional brain volume and measures of RRB. Measures of RRB included Section C of the Autism Diagnostic Interview – Revised (ADI-R), as well Repetitive Behavior Scale – Revised (RBS-R) total score and subsection scores. Significant FDR corrected p-values are bolded and indicated by *****p<0.05, ******p<0.01, *******p<0.001.

**Table S4:** Spearman correlations for fractional anisotropy (uncorrected) in basal ganglia and cerebellar white matter tracts

| **Tract FA** | **ADI-R Section C** | | **RBS-R Total** | | **RBS-R Stereotyped** | | **RBS-R**  **Self-Injury** | | **RBS-R Compulsive** | | **RBS-R Ritual** | | **RBS-R Sameness** | | **RBS-R Restricted Interests** | | **SCQ** | |
| --- | --- | --- | --- | --- | --- | --- | --- | --- | --- | --- | --- | --- | --- | --- | --- | --- | --- | --- |
|  | p_raw_ | p_FDR_ | p_raw_ | p_FDR_ | p_raw_ | p_FDR_ | p_raw_ | p_FDR_ | p_raw_ | p_FDR_ | p_raw_ | p_FDR_ | p_raw_ | p_FDR_ | p_raw_ | p_FDR_ | p_raw_ | p_FDR_ |
| DLPFC to Caudate (L) | 0.771 | 0.964 | <0.001 | *****<0.001** | <0.001 | *****<0.001** | 0.015 | 0.152 | 0.001 | ***0.014** | <0.001 | *****<0.001** | <0.001 | *****<0.001** | <0.001 | *****<0.001** | <0.001 | ****0.002** |
| DLPFC to Caudate (R) | 0.243 | 0.811 | <0.001 | ****0.002** | <0.001 | *****<0.001** | 0.065 | 0.327 | 0.036 | 0.182 | 0.004 | ***0.020** | 0.001 | ****0.007** | <0.001 | *****<0.001** | 0.005 | ***0.025** |
| M1U to Putamen (L) | 0.975 | 0.975 | 0.622 | 0.758 | 0.600 | 0.600 | 0.299 | 0.599 | 0.810 | 0.810 | 0.552 | 0.789 | 0.537 | 0.671 | 0.838 | 0.929 | 0.904 | 0.904 |
| M1U to Putamen (R) | 0.549 | 0.937 | 0.291 | 0.416 | 0.090 | 0.150 | 0.566 | 0.713 | 0.466 | 0.665 | 0.314 | 0.629 | 0.269 | 0.469 | 0.499 | 0.624 | 0.741 | 0.823 |
| SCP to M1U (L) | 0.402 | 0.937 | 0.051 | 0.171 | 0.035 | 0.088 | 0.175 | 0.584 | 0.152 | 0.505 | 0.484 | 0.789 | 0.291 | 0.469 | 0.019 | 0.064 | 0.050 | 0.166 |
| SCP to M1U (R) | 0.682 | 0.964 | 0.682 | 0.758 | 0.084 | 0.150 | 0.741 | 0.824 | 0.731 | 0.810 | 0.990 | 0.990 | 0.864 | 0.960 | 0.302 | 0.431 | 0.349 | 0.498 |
| SN to Putamen (L) | 0.081 | 0.807 | 0.234 | 0.390 | 0.183 | 0.229 | 0.922 | 0.922 | 0.321 | 0.642 | 0.713 | 0.891 | 0.328 | 0.469 | 0.114 | 0.191 | 0.213 | 0.451 |
| SN to Putamen (R) | 0.216 | 0.811 | 0.918 | 0.918 | 0.570 | 0.600 | 0.570 | 0.713 | 0.752 | 0.810 | 0.802 | 0.891 | 0.983 | 0.983 | 0.929 | 0.929 | 0.605 | 0.756 |
| GPe to STN (L) | 0.934 | 0.975 | 0.111 | 0.251 | 0.123 | 0.175 | 0.523 | 0.713 | 0.410 | 0.665 | 0.110 | 0.274 | 0.312 | 0.469 | 0.058 | 0.144 | 0.271 | 0.451 |
| GPe to STN (R) | 0.562 | 0.937 | 0.126 | 0.251 | 0.035 | 0.088 | 0.298 | 0.599 | 0.237 | 0.592 | 0.040 | 0.134 | 0.212 | 0.469 | 0.072 | 0.144 | 0.255 | 0.451 |

**Table S4 legend:** Raw and FDR corrected p-values from Spearman correlations between uncorrected fractional anisotropy (FA) in basal ganglia and cerebellar tracts and behavioral measures. Measures of RRB included Section C of the Autism Diagnostic Interview – Revised (ADI-R), as well Repetitive Behavior Scale – Revised (RBS-R) total score and subsection scores. The Social Communication Questionnaire (SCQ) measures social and communication behaviors. Significant FDR corrected p-values are bolded and indicated by *****p<0.05, ******p<0.01, *******p<0.001.

**Table S5: Pearson correlations between uncorrected FA and FA_T_**

| **TRACT** | **UNCORRECTED FA**  **MEAN ± STDEV** | **FA_T_**  **MEAN ± STDEV** | **R** | **p-value** |
| --- | --- | --- | --- | --- |
| DLPFC to Caudate (L) | 0.446 ± 0.017 | 0.363 ± 0.025 | 0.767 | *****<0.001** |
| DLPFC to Caudate (R) | 0.412 ± 0.017 | 0.314 ± 0.024 | 0.771 | *****<0.001** |
| M1U to Putamen (L) | 0.405 ± 0.018 | 0.510 ± 0.045 | 0.293 | *****<0.001** |
| M1U to Putamen (R) | 0.400 ± 0.020 | 0.514 ± 0.046 | 0.319 | *****<0.001** |
| SCP to M1U (L) | 0.536 ± 0.015 | 0.350 ± 0.028 | 0.525 | *****<0.001** |
| SCP to M1U (R) | 0.522 ± 0.017 | 0.348 ± 0.028 | 0.637 | *****<0.001** |
| SN to Putamen (L) | 0.420 ± 0.018 | 0.464 ± 0.026 | 0.413 | *****<0.001** |
| SN to Putamen (R) | 0.407 ± 0.019 | 0.445 ± 0.028 | 0.368 | *****<0.001** |
| GPe to STN (L) | 0.562 ± 0.023 | 0.348 ± 0.026 | 0.552 | *****<0.001** |
| GPe to STN (R) | 0.579 ± 0.026 | 0.335 ± 0.028 | 0.417 | *****<0.001** |

**Table S5 legend:** Means and standard deviations (all participants) for the measures of uncorrected fractional anisotropy (FA) and free-water corrected fractional anisotropy (FA_T_) in basal ganglia and cerebellar white matter tracts. The corresponding Pearson correlation coefficients between those two metrics and associated p-values are reported for each tract. Significant FDR corrected p-values are bolded and indicated by *****p<0.05, ******p<0.01, *******p<0.001.
